# A fully protected hydrogenase/polymer-based bioanode for high-performance hydrogen/glucose biofuel cells

**DOI:** 10.1038/s41467-018-06106-3

**Published:** 2018-09-10

**Authors:** Adrian Ruff, Julian Szczesny, Nikola Marković, Felipe Conzuelo, Sónia Zacarias, Inês A. C. Pereira, Wolfgang Lubitz, Wolfgang Schuhmann

**Affiliations:** 1 Analytical Chemistry - Center for Electrochemical Sciences (CES), Ruhr-Universität Bochum, Universitätsstr. 150, Bochum, D-44780 Germany; 2 Instituto de Tecnologia Química e Biológica António Xavier, Universidade Nova de Lisboa, Oeiras, 2780-157 Portugal; 3 Max-Planck-Institut für Chemische Energiekonversion, Stiftstrasse 34–36, Mülheim an der Ruhr, 45470 Germany

## Abstract

Hydrogenases with Ni- and/or Fe-based active sites are highly active hydrogen oxidation catalysts with activities similar to those of noble metal catalysts. However, the activity is connected to a sensitivity towards high-potential deactivation and oxygen damage. Here we report a fully protected polymer multilayer/hydrogenase-based bioanode in which the sensitive hydrogen oxidation catalyst is protected from high-potential deactivation and from oxygen damage by using a polymer multilayer architecture. The active catalyst is embedded in a low-potential polymer (protection from high-potential deactivation) and covered with a polymer-supported bienzymatic oxygen removal system. In contrast to previously reported polymer-based protection systems, the proposed strategy fully decouples the hydrogenase reaction form the protection process. Incorporation of the bioanode into a hydrogen/glucose biofuel cell provides a benchmark open circuit voltage of 1.15 V and power densities of up to 530 µW cm^−2^ at 0.85 V.

## Abstract (web summary)

Hydrogenases are promising alternatives to noble metal-based catalysts for hydrogen oxidation. Here the authors fully protect air-sensitive hydrogenases from high potential and oxygen damage using a polymer multilayer bioanode in a biofuel cell that delivers a benchmark open circuit voltage.

## Introduction

Hydrogenases are nature´s highly efficient biocatalysts for the reversible conversion of H_2_ into protons^1^. Their rate constants for H_2_ oxidation are similar to those of scarce and costly noble-metal-based catalysts like Pt^2,3^. In the late 1970s they were proposed as promising alternatives for the fabrication of high current-density (*J*) H_2_-oxidizing anodes either in a direct electron transfer (DET) or a mediated electron transfer (MET) regime^4^. In combination with an O_2_-reducing biocatalyst, typically the multicopper enzymes bilirubin oxidase or laccase, biofuel cells (BFCs) with remarkably high open circuit voltage (OCV) of up to 1.17 V^5^ and with maximum power densities (*P*) ranging from 1.7^6^ to even 8.4 mW cm^−2^ when operated in gas breathing mode^7^ and in a DET configuration were constructed^8–11^. However, their intrinsic sensitivity toward O_2_ and high anodic potentials hampers the application of these biocatalysts in technologically relevant devices because of a fast deactivation under operating conditions^8–10^. Strategies to enhance the O_2_ tolerance of the immobilized biocatalyst include the use of porous electrodes (blocking of O_2_ diffusion) or the use of specific heterotrimeric membrane-bound [NiFe]-hydrogenases embedded in quinone containing lipid bilayers, as demonstrated by the Armstrong^12^ and Jeuken^13^ groups, respectively. The latter strategy also ensures protection from high potential deactivation even under substrate limiting conditions^13^. Also a possible reactivation of the deactivated enzyme under H_2_ atmosphere by reduced viologen species was described^14^.

An elegant and efficient way to protect hydrogenases from O_2_ and from high potential deactivation is the incorporation of the enzyme into specifically designed low-potential viologen-modified redox polymers, as it was demonstrated for various O_2_ sensitive hydrogenases ([NiFe]^15^, [FeFe]^16^, and [NiFeSe]^17^). The low-potential redox polymer does not only act as an immobilization matrix and an electron relay between the electrode and the biocatalyst, but it also acts as a Nernst buffer system that prevents high-potential deactivation. Simultaneously, O_2_ is eliminated by reduction at reduced viologen moieties at the polymer/electrolyte interface. The electrons for the oxygen reduction reaction (ORR) are delivered from the biocatalyst itself by transferring the electrons from H_2_ oxidation to oxidized viologen species^15,18^. Although this strategy allows for fabrication of even membrane-free H_2_/O_2_ BFCs^15^, thick films and thus rather large amounts of the biocatalyst have to be employed to ensure a discrete separation of the H_2_ oxidation layer close to the electrode surface and the O_2_ protection layer at the polymer/electrolyte interface (note that in thin films, the enzyme is deactivated rapidly by O_2_; however, the reduced low-potential polymer matrix may reactivate the deactivated enzyme even in the absence of H_2_ as it was demonstrated for a [NiFeSe] hydrogenase^17;^ for a more detailed description of the protection mechanism the reader is referred to ref. ^18^). Consequently, in the presence of O_2_ parts of the turnover of the H_2_ oxidizing catalysts are wasted for the protection of the catalytic layer and do not contribute to the H_2_ oxidation current, i.e., electrons from the outer layer are transferred to O_2_ and not to the electrode. This effect is illustrated in chronoamperometric experiments conducted under turnover conditions upon addition of O_2_ to the gas feed: a reversible decrease of the oxidation current due to consumption of electrons for the ORR is observed^15–17^. Evidently, a complete protection of the active hydrogenase layer without consuming electrons from H_2_ oxidation would be beneficial and additional protection strategies are desired.

Enzymatic O_2_ removal systems based on an oxidase (e.g., glucose oxidase) and catalase (CAT) are well known to ensure anaerobic conditions typically in solution^19–22^. Within each catalytic cycle, ½O_2_ molecules are removed in the presence of an electron donor, i.e., glucose in the case of glucose oxidase (GOx) (Eq. (1–3)).

1 $$ {{\mathrm{Glucose}}\;{\mathrm{oxidase}}\;{\mathrm{reaction:}}\;{\mathrm{glucose + O}}_{\mathrm{2}} \to {\mathrm{gluconolactone + H}}_{\mathrm{2}}{\mathrm{O}}_{\mathrm{2}}}$$

2 $${\mathrm{Catalase}}\;{\mathrm{reaction}}\;\left( {{\mathrm{disproportionation}}} \right){\mathrm{:}}\;{\mathrm{H}}_{\mathrm{2}}{\mathrm{O}}_{\mathrm{2}} \to {\mathrm{H}}_{\mathrm{2}}{\mathrm{O + \raise.5ex\hbox{$\scriptstyle 1$}\kern-.1em/ \kern-.15em\lower.25ex\hbox{$\scriptstyle 2$} O}}_{\mathrm{2}}$$

3 $${\mathrm{Net}}\;{\mathrm{reaction:}}\;{\mathrm{glucose + \raise.5ex\hbox{$\scriptstyle 1$}\kern-.1em/ \kern-.15em\lower.25ex\hbox{$\scriptstyle 2$}O}}_{\mathrm{2}} \to {\mathrm{gluconolactone + H}}_{\mathrm{2}}{\mathrm{O}}.$$

This enzyme cascade removes not only oxygen but also harmful H_2_O_2_ (Eq. (2)) that might be produced by an incomplete reduction of O_2_ at the low-potential viologen mediator. Recently, we have shown that immobilization of lactate oxidase (O_2_ removal element when lactate is present) and catalase in a redox-silent polymer matrix on top of a GOx-based sensing layer in which the biocatalyst was wired via a low potential, O_2_-reducing toluidine blue-modified redox polymer leads to a O_2_-insensitive GOx-based glucose-converting bioanode, which could be operated under ambient conditions^23^.

In addition to the highly efficient hydrogenase-based bioanodes, stable and highly active biocatalysts with low overpotentials for O_2_ reduction are also required for the biocathode reaction. Although bilirubin oxidase^24^ and laccase^24,25^ show high turnover rates for the ORR, their low intrinsic stability against halides^26–31^ and deactivation by H_2_O_2_^32–34^ may hamper their use in high-performance and technologically relevant BFCs.

Horseradish peroxidase (HRP) is a robust enzyme that is readily available and catalyzes the 2e^−^/2H^+^ reduction of H_2_O_2_ to water^35,36^. The oxidant H_2_O_2_ can be generated in situ e.g., most frequently by GOx^37–42^ or other oxidases, like pyranose oxidase^43^ in the presence of glucose. Moreover, HRP can be electrically wired at extraordinary high potentials of +860 mV vs. SHE via the high potential iron-oxo complex Fe^IV^ = O,heme^+^·(compound I)^35,36^ in DET regime using specifically modified carbon electrodes, i.e., temperature-treated spectrographic graphite electrodes^44,45^ or carbon nanotube (CNT)-modified graphite electrodes^37,38,46^. The low overpotential for the overall 4e^−^/4 H^+^ reduction of O_2_ to water (via GOx and HRP) makes this enzyme cascade a promising alternative to the highly active but also sensitive multicopper oxidases for high current-density biocathodes^39–41^.

Here, we report the design of a fully protected polymer multilayer-based hydrogenase bioanode combined with an oxidase/HRP biocathode for the fabrication of a H_2_-powered BFC that consumes H_2_O_2_ as the oxidant which is generated in situ from O_2_ by an oxidase and in the presence of glucose to keep the concentration of harmful H_2_O_2_ low. The device shows an extraordinary high OCV and remarkable current densities.

## Results

### Electrode architectures and concept

**Fig. 1:**
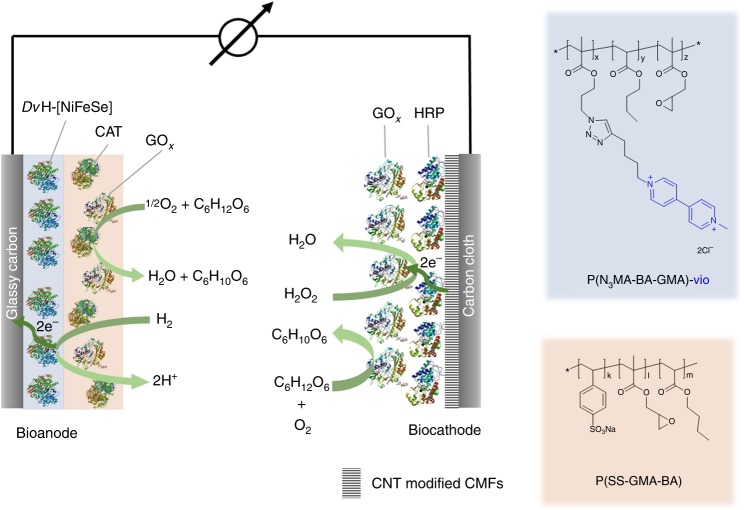
Schematic of the proposed hydrogenase/horseradish peroxidase biofuel cell. The bioanode consists of a polymer double-layer system comprising an underlying layer composed of the [NiFe] or [NiFeSe] hydrogenase from *Desulfovibrio vulgaris* Miyazaki F (*Dv*MF-[NiFe]) and from *Desulfovibrio vulgaris* Hildenborough (*Dv*H-[NiFeSe]) integrated into the viologen-modified polymer P(N_3_MA-BA-GMA)-vio (poly(3-azido-propyl methacrylate-*co*-butyl acrylate-*co*-glycidyl methacrylate)-viologen (the viologen moieties are depicted in blue) and a protection top layer consisting of a bienzymatic system that contains an oxidase (glucose oxidase, GOx, or pyranose oxidase, Py_2_Ox) and catalase (CAT) embedded in the redox-silent polymer P(SS-GMA-BA) (poly(4-styrene sulfonate-*co*-glycidyl methacrylate-*co*-butyl acrylate)). Oxygen is removed by converting glucose to gluconolactone by the concomitant reduction of O_2_ to H_2_O_2_. The biocathode is built on a carbon cloth electrode that was modified with carbon microfibers (CMFs) that were decorated with carbon nanotubes (CNTs). The latter ensure wiring of the horseradish peroxidase (HRP) via the iron-oxo complex compound I. The cathode was first modified with pyrene butyric acid (hydrophilization) and then via a sequential drop cast process with a HRP layer followed by a top layer containing the oxidase (GOx or Py_2_Ox) that ensures the in situ H_2_O_2_ formation. Note that for clarity only the combination of a *Dv*H-[NiFeSe]/GOx/CAT and GOx/HRP based electrodes are shown in the scheme

The architecture of the proposed O_2_-protected hydrogenase bioanode is based on a polymer multilayer architecture comprising an active H_2_ oxidation layer in which a hydrogenase, i.e., [NiFe] hydrogenase from *Desulfovibrio vulgaris* Miyazaki F (*Dv*MF-[NiFe])^47^ or [NiFeSe] hydrogenase from *Desulfovibrio vulgaris* Hildenborough (*Dv*H-[NiFeSe])^48^, is wired via a low-potential viologen-modified polymer, i.e., the polymer matrix P(N_3_MA-BA-GMA)-vio (poly(3-azido-propyl methacrylate-*co*-butyl acrylate-*co*-glycidyl methacrylate)-viologen, Fig. 1, right)^17^, and an O_2_ removing top layer composed of an oxidase and catalase entrapped in the redox-silent hydrophilic polymer matrix P(SS-GMA-BA) (poly(4-styrenesulfonate-*co*-glycidyl methacrylate-*co*-butyl acrylate)^49^, Fig. 1, right). The bioanode configuration and the proposed protection mechanism for the active layer is illustrated in Fig. 1 (left). The oxidase/catalase O_2_ removal system is fueled by glucose which simultaneously acts as the reactant for the in situ generation of the oxidant H_2_O_2_ at the oxidase/HRP-based biocathode. H_2_O_2_ will only be formed by the oxidase in the presence of O_2_. Consequently, also for the proposed H_2_/glucose fuel cell, protection of the bioanode from O_2_ is indispensably required. Nanostructured electrodes are based on a high surface area carbon cloth that is modified with CNT-decorated carbon microfibers (CMFs), introduced for the wiring of HRP. The carbon nanostructures are a prerequisite for the productive wiring of HRP via the high-potential iron-oxo complex compound I. The proposed system provides full protection of the hydrogenases by combining O_2_ protection by means of the bienzymatic O_2_ removal system in the outer layer with protection from high-potential deactivation by means of the viologen-modified polymer that acts as Nernst buffer and should ensure a constant power output even in the presence of oxygen.

### Bioanode

The efficiency of the polymer-supported oxidase/catalase O_2_ removal system (Eq. (1–3)) for the protection of the active polymer/hydrogenase layer was first tested with bare Pt electrodes that were coated with a GOx/CAT/P(SS-GMA-BA) layer (prepared according to ref. ^50^). The Pt electrode was polarized at a potential of +10 mV vs. SHE to induce ORR at the electrode surface, as can be seen from a constant cathodic current flow (Supplementary Fig. 1a). Upon addition of glucose (50 mM) the current dropped to almost zero due to the efficient removal of O_2_ at the interface of the protection layer. Since the polymer-bound viologen itself is a catalyst for the ORR^15^, the signal of the viologen moiety in the cyclic voltammograms can be used to evaluate the efficiency of the O_2_ removal system in the double-layer configuration. For this, pristine P(N_3_MA-BA-GMA)-vio films were coated with a large excess of the GOx/CAT/P(SS-GMA-BA) mixture in a drop cast process to form a P(N_3_MA-BA-GMA)-vio//P(SS-GMA-BA)/GOx/CAT double-layer system (note that for all experiments a large excess of the second layer with respect to polymer mass was used to ensure the full coverage of the active hydrogenase layer, for composition and polymer/enzyme ratios see Methods part). Cyclic voltammograms in phosphate buffer (0.1 M, pH 7.4) containing glucose (50 mM) under argon (Supplementary Fig. 1b, black dashed line) and under air (red line) show the unchanged reversible signal of the polymer-bound viologen moiety. A significant O_2_ reduction via the low potential viologen based mediator^15^, as it was observed for voltammograms recorded without glucose in solution (blue line), was absent indicating that O_2_ is fully removed in the top layer. This also indicates that both layers do not intermix significantly during rehydration since no direct exposure of the viologen-modified polymer/hydrogenase layer to the bulk electrolyte is detected. In contrast to the experiments in quiescent solutions, slightly enhanced cathodic currents were observed due to enhanced mass transport, when a mixture of 5% O_2_ and 95% Ar is purged through the electrolyte. Trace amounts of O_2_ seem to reach the underlying active layer under these conditions (Supplementary Fig. 1c). However, under H_2_ turnover conditions, these trace amounts of O_2_ are reduced at the viologen moiety and hence will not significantly affect the performance of the bioanode.

**Fig. 2:**
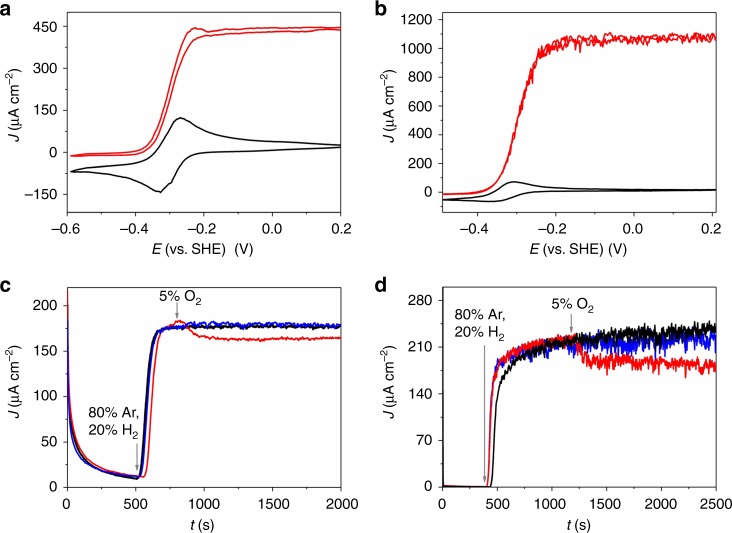
Electrochemical characterization of the bioanode. Cyclic voltammetry (**a**, **b**) and chronoamperometry (**c**, **d**) of glassy carbon electrodes modified with the polymer multilayer system comprising an underlying P(N_3_MA-BA-GMA)-vio/hydrogenase layer (drop cast, **a**, **c**: [NiFe] hydrogenase from *Desulfovibrio vulgaris* Miyazaki F, *Dv*MF-[NiFe], **b**, **d**: [NiFeSe] hydrogenase from *Desulfovibrio vulgaris* Hildenborough, *Dv*H-[NiFeSe]) and an outer protection layer based on P(SS-GMA-BA)/GOx/CAT (drop cast). Working electrolyte: 0.1 M phosphate buffer (pH 7.4), **a** and **b**: scan rate: 10 mV s^−1^; black traces: 100% argon, red traces: 100% H_2_; **c**, **d**: applied potential: +160 mV vs. standard hydrogen electrode (SHE); black traces: 80% Ar and 20% H_2_; red traces: 75% Ar, 20% H_2_ and 5% O_2_ without glucose, blue traces: 75% Ar, 20% H_2_ and 5% O_2_ with glucose in solution (50 mM). Gray arrows in **c** and **d** indicate a change of the gas feed composition. P(N_3_MA-BA-GMA)-vio = poly(3-azido-propyl methacrylate-*co*-butyl acrylate-*co*-glycidyl methacrylate)-viologen; P(SS-GMA-BA) = poly(4-sytyrenesulfonate-*co*-glycidyl methacrylate-*co*-butyl acrylate); GOx; glucose oxidase, CAT; catalase

Cyclic voltammograms (Fig. 2a, b and Supplementary Fig. 2a) recorded with P(N_3_MA-BA-GMA)-vio/hydrogenase films drop cast onto glassy carbon electrodes and covered with P(SS-GMA-BA)/GOx/CAT layers under Ar and H_2_ atmosphere show pronounced catalytic waves revealing half wave potentials (*Dv*MF-[NiFe]/P(N_3_MA-BA-GMA)-vio: ≈ −0.27 V vs. SHE; *Dv*H-[NiFeSe]/P(N_3_MA-BA-GMA)-vio: ≈ −0.32 V vs. SHE) that match nicely the redox potential of the redox polymer (−0.3 V vs. SHE), indicating a successful wiring of the hydrogenase in a MET regime. The current responses of the bioanodes are similar to previously reported polymer/hydrogenase-based bioanodes^15,17^.

The mass transport of H_2_ is obviously only slightly hampered by the protection layer demonstrated by the marginally higher steady state currents for the single layer system P(N_3_MA-BA-GMA)-vio/*Dv*MF-[NiFe] under H_2_ turnover conditions (cf. Supplementary Fig. 2a, b). The catalytic current in the double-layer system is limited by the H_2_ concentration with a linear current increase up to 40% H_2_ (Supplementary Fig. 2c, d). This behavior corresponds to regime III or case III following the notation described in refs. ^18,51^, respectively. Note that for this behavior a current decrease is expected upon O_2_ addition if no additional protection system is used^15,18^.

Chronoamperometry in the presence of glucose with *Dv*MF-[NiFe] or *Dv*H-[NiFeSe] hydrogenase-bioanodes coated with the protection layer show constant steady state H_2_ oxidation currents under 20%H_2_/ 80%Ar (Fig. 2c, d, black traces) and 5% O_2_/20%H_2_/ 80%Ar (blue lines) indicating that only insignificant numbers of reduced viologen units are involved in the O_2_ protection by the polymer multilayer system. In contrast, in the absence of glucose (Fig. 2c, d, red traces), the anodic current of the double-layer system decreases to a lower steady state value due to the continuous consumption of electrons from H_2_ oxidation by O_2_ reduction at the viologen-modified polymer matrix in analogy to electrodes coated only with a polymer/hydrogenase reaction layer^15–17^.

For high H_2_ concentrations the *Dv*H-[NiFeSe] based electrodes show higher *J*-values compared to the *Dv*MF-[NiFe] (cf. Fig. 2a, b) which is in line with our previous results^15,17^. However, for lower H_2_ contents the difference seems to be lower (cf. Fig. 2c, d) which may be related to the fact that the currents are limited by the mass transport at these low H_2_ levels.

**Fig. 3:**
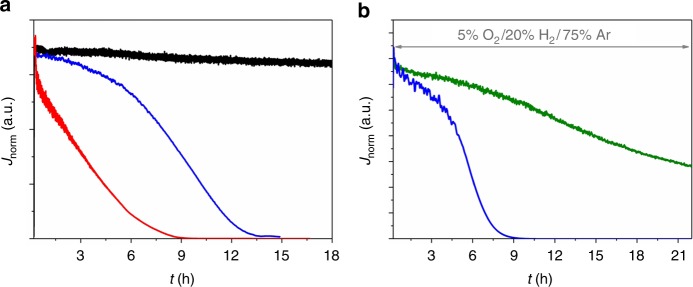
Long-term stability of the polymer multilayer-based bioanodes. Chronoamperometric long-term stability measurements of the double-layer bioanodes in 0.1 M phosphate buffer, pH 7.4 at an applied potential of +160 mV vs. standard hydrogen electrode (SHE) under turnover conditions and in the presence of O_2_. **a** Black trace: P(N_3_MA-BA-GMA)-vio/*Dv*MF-[NiFe]//P(SS-GMA-BA)/GOx/CAT system with 20% H_2_/80% Ar bubbling through the cell and in the absence of glucose; red trace: single P(N_3_MA-BA-GMA)-vio/*Dv*MF-[NiFe] layer, 5% O_2_/20% H_2_/75% Ar; blue trace: P(N_3_MA-BA-GMA)-vio/*Dv*MF-[NiFe]//P(SS-GMA-BA)/GOx/CAT system with 5% O_2_/20% H_2_/75% Ar bubbling through the cell and 100 mM glucose; the same enzyme batch was used for all three experiments. **b** Comparison between the protection systems based on GOx (P(N_3_MA-BA-GMA)-vio/*Dv*MF-[NiFe]//P(SS-GMA-BA)/GOx/CAT, blue line) and Py_2_Ox (P(N_3_MA-BA-GMA)-vio/*Dv*MF-[NiFe]//P(SS-GMA-BA)/Py_2_Ox/CAT, green line) with 5% O_2_/20% H_2_/75% Ar bubbling through the electrolyte and with 50 mM glucose in solution; the same enzyme batch was used for both measurements. For comparative purposes the current densities were normalized to the current flow at 100% H_2_ (*J*_norm_) to eliminate variations in *J* (for absolute current values (*I*) see Supplementary Fig. 3). P(N_3_MA-BA-GMA)-vio = poly(3-azido-propyl methacrylate-*co*-butyl acrylate-*co-*glycidyl methacrylate)-viologen; P(SS-GMA-BA) = poly(4-sytyrenesulfonate-*co*-glycidyl methacrylate-*co*-butyl acrylate); GOx; glucose oxidase, CAT; catalase, *Dv*MF-[NiFe]; [NiFe] hydrogenase from *Desulfovibrio vulgaris* Miyazaki F

Polymer multilayer modified bioanodes based on the *Dv*MF-[NiFe] hydrogenase show rather constant currents in long-term chronoamperometric experiments under H_2_ atmosphere. Only a slight decrease of the H_2_ oxidation current is observed over a period of 18 h (Fig. 3a, black line, for absolute currents see Supplementary Fig. 3a), indicating that the biocatalyst and the mediator (see also Supplementary Fig. 4a) are stable within the timescale of the experiment. In the absence of the protection system and in the presence of O_2_ a rather fast linear decrease of the oxidation currents to background values within 9 h was observed since the oxygen front reaches the reaction layer within the course of the experiment (Fig. 3a, red line, Supplementary Fig. 3a, red line). This does not only lead to a deactivation of the hydrogenases but also diminishes the signal of the current amplitude of the polymer-bound viologen units in voltammograms recorded after the long-term experiment (Supplementary Fig. 4b) most likely due to a partial decomposition of the mediator under aerobic conditions (formation of H_2_O_2_ by the viologen).

In the case of the double-layer system in the presence of glucose and upon addition of 5% O_2_ to the gas feed only a rather weak decrease of the H_2_ oxidation current was observed within the first hours (0–6 h) of the experiments (Fig. 3a and Supplementary Fig. 3a, blue lines). At *t* > 6 h, a sudden current drop occurred and H_2_ oxidation was finally stopped at *t* > 13 h. This sudden decrease of the current is striking and differs from the behavior that could be expected from the linear decay for the system without protection. Cyclic voltammograms recorded with electrodes that were transferred into a fresh electrolyte still show the less pronounced redox waves of the polymer-bound viologen units (Supplementary Fig. 4c). Hence, we conclude that the biocatalyst was irreversibly deactivated under these conditions even in the presence of the O_2_ removal system. Moreover, reactivation via the reduced viologen moieties as it was observed for thin P(N_3_MA-BA-GMA)-vio/*Dv*H-[NiFeSe] (see ref. ^17^) and P(N_3_MA-BA-GMA)-vio/*Dv*MF-[NiFe] films (Supplementary Fig. 5) deactivated by O_2_ was not possible. Evidently, there must be an additional reason for the deactivation of the polymer entrapped hydrogenase, which is not due to the presence of O_2_. The pH value of the buffer solution changed during catalysis from 7.4 at the beginning to 4.4 at the end of the experiment due to the formation of gluconolactone which is readily hydrolyzed to gluconic acid. In combination with the protons generated by the conversion of H_2_ at the bioanode, the local concentration of H^+^ within the polymer/enzyme film exceeds the buffer capacity in the solvated hydrogel and the pH drops to a critical value at which the enzyme is disintegrated.

Since Eq. (1–3) hold true for any oxidase, we exploited the use of other glucose-converting systems as protection layer, e.g., pyranose 2-oxidase (Py_2_Ox). This particular enzyme catalyzes the oxidation of glucose to 2-dehydro-d-glucose. This rather stable and inert ketone is not prone to undergo hydrolysis in water. Consequently, the pH value of the solution does not change during catalysis as previously reported for Py_2_Ox/CAT in solution^19^. Moreover, in situ generation of H_2_O_2_ by Py_2_Ox was successfully coupled to HRP^43^ and is hence suitable for the fabrication of the proposed biocathode.

The performance of Py_2_Ox/CAT to remove O_2_ is lower than that of GOx/CAT due to the lower activity of Py_2_Ox and O_2_ removal is only possible up to 3% O_2_ (Supplementary Fig. 6). However, the chronoamperometric long-term evaluation shows a constant current decay (Fig. 3b, green line). Cyclic voltammograms recorded under argon after the chronoamperometric experiment show slightly decreased signals of the viologen units indicating that also the polymer matrix, although to a lower extent when compared to the GOx-based system, is affected under these conditions (Supplementary Fig. 7). Hence, not only enzyme deactivation but also polymer degradation may contribute to the observed current decrease.

### Biocathode

For the wiring of HRP, a carbon cloth decorated with CNT-modified CMFs was used as base electrode material^37,38^. The carbon cloth was first modified with CMFs in an Fe catalyzed chemical vapor deposition (CVD) process, followed by a second Fe catalyzed CVD process, in which the CMFs were decorated with CNTs (Supplementary Fig. 8).

The CMFs-modified carbon cloth ensures a high surface area, while the CNTs allow for the wiring of compound I within the oxidized HRP at high positive potentials^46^. Supplementary Fig. 9 shows the cyclic voltammogram of a drop cast HRP-modified CNT/CMF-carbon cloth electrode in the absence (black line) and presence of 2 mM H_2_O_2_ (red line). H_2_O_2_ reduction at compound I is counterbalanced by direct H_2_O_2_ oxidation at the carbon electrode. Thus, H_2_O_2_ reduction becomes visible at about +0.75 V vs. SHE which is more positive than the BOD catalyzed O_2_ reduction potential of up to 0.67 V vs. SHE^31^. As compared to O_2_ reducing biocathodes that catalyze the full 4e^−^/4 H^+^ reduction of O_2_ to water, the high potential of this biocathode must be paid with the loss of two electrons in the cathodic reaction. However, since the size of this electrode is easily scalable, the 2e^−^ reduction can be fully compensated using an oversized biocathode (with respect to the bioanode).

**Fig. 4:**
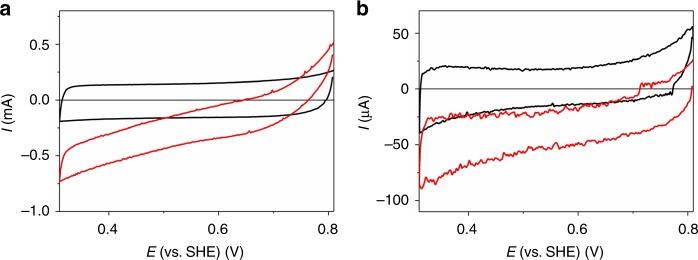
Voltammetric characterization of the biocathode. **a** Cyclic voltammograms of a glucose oxidase/horseradish peroxidase modified carbon nanotube (CNT)/carbon microfiber (CMF)-carbon cloth electrode in presence of 50 mM glucose under argon (black line) and O_2_ (red line) atmosphere; scan rate: 5 mV s^−1^. **b** Cyclic voltammograms of a pyranose oxidase/horseradish peroxidase modified CNT/CMF-carbon cloth electrode with O_2_ bubbling through the electrolyte in absence (black line) and presence (red line) of glucose (1 mM). The geometrical surface area of the carbon cloth-based electrode was 1 cm^2^ thus the current densities equal the absolute currents. However, it should be noted that the use of the geometrical surface is for the calculation of *J* is a rough approximation since the electrodes reveal a rather porous 3D structure. All voltammetric scans were conducted in 0.1 M phosphate buffer, pH 7.4. SHE; standard hydrogen electrode

The immobilization of the oxidase for the in situ generation of H_2_O_2_ was performed in a second drop cast process on top of the HRP layer. Cyclic voltammograms of GOx/HRP (A) or Py_2_Ox/HRP (B) modified CNT/CMF-carbon cloth electrodes recorded under argon (black line) and under O_2_ (red line) in 0.1 M PB (pH 7.4) containing 50 mM glucose are shown in Fig. 4. Obviously, both enzymes are able to produce substantial amounts of the oxidant H_2_O_2_ as indicated by the pronounced catalytic waves under turnover conditions (red lines, note that the current response of the carbon cloth-based electrodes is similar to our previously described systems based on CNT/CMF modified carbon rods^37,38^). While the absolute currents are lower than those obtained for electrodes equipped with only HRP in the presence of 2 mM H_2_O_2_, the potential at which visible H_2_O_2_ reduction occurs does not significantly change for the oxidase/HRP system. Moreover, by varying the glucose concentration, the current output at the biocathode can be controlled and adjusted to that of the bioanode (Supplementary Fig. 10a). In addition, chronoamperometric experiments conducted with the GOx/HRP system does not lose its activity over a period of 6 h (Supplementary Fig. 10b).

### Biofuel cell

**Fig. 5:**
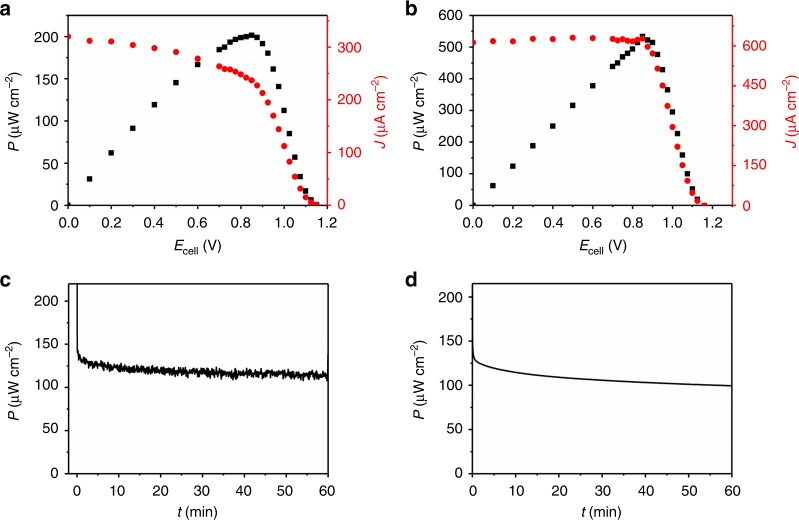
Characterization of the hydrogen/glucose biofuel cell. Performance (**a**, **b**) and stability (**c**, **d**) of the H_2_/glucose(H_2_O_2_)-powered hydrogenase(H_2_ase)/ horseradish peroxidase (HRP)-based biofuel cells in 0.1 M phosphate buffer (pH 7.4) and in a two-compartment cell with the polymer double-layer bioanode (P(N_3_MA-BA-GMA)-vio/H_2_ase//P(SS-GMA-BA)/Py_2_Ox/CAT) and the Py_2_Ox/HRP/CNT/CMF-carbon cloth biocathode. **a**: H_2_ase = *Dv*MF-[NiFe], 1 mM glucose in both compartments; OCV = 1.15 V; *J* = 240 µA cm^−2^ and *P*_max_ = 200 µW cm^−2^ at 0.85 V. **b** H_2_ase = *Dv*H-[NiFeSe], 3 mM glucose in both compartments. OCV = 1.15 V; *J* = 630 µA cm^−2^ and *P*_max_ = 530 µW cm^−2^ at 0.85 V. **c**, **d** Operational stability at a constant load of 0.8 V of the *Dv*MF-[NiFe] (**c**) and the *Dv*H-[NiFeSe] (**d**) based BFC; 3 mM glucose was used in the bioanode and the biocathode compartment. **a**–**d** Bioanode compartment = 97% H_2_/3% Ar and biocathode compartment = 50% O_2_/50% Ar. *J* and *P* were referenced to the geometrical surface are of the glassy carbon-based anode (0.07 cm^2^); note that different electrodes were used for the experiments depicted in **a**/**c** and **b**/**d**, respectively. P(N_3_MA-BA-GMA)-vio = poly(3-azido-propyl methacrylate-*co*-butyl acrylate-*co*-glycidyl methacrylate)-viologen; P(SS-GMA-BA) = poly(4-sytyrenesulfonate-*co*-glycidyl methacrylate-*co*-butyl acrylate); Py_2_Ox; pyranose oxidase, CAT; catalase, *Dv*MF-[NiFe]; [NiFe] hydrogenase from *Desulfovibrio vulgaris* Miyazaki F, *Dv*H-[NiFeSe]; [NiFeSe] hydrogenase from *Desulfovibrio vulgaris* Hildenborough

The performance of a BFC composed of the double-layer-protected hydrogenase bioanode and the oxidase/HRP biocathode was evaluated in a two-compartment cell separated by a Nafion membrane. A BFC comprising a *Dv*MF-[NiFe]/P(N_3_MA-BA-GMA)-vio//Py_2_Ox/CAT/P(SS-GMA-BA) bioanode and a Py_2_Ox/HRP/CNT-CMF-carbon cloth biocathode exhibits an OCV of about 1.15 V (Fig. 5a), which is significantly larger than the OCV of reported polymer-based [NiFe] (0.95 V)^15^ or [FeFe] (1.08 V)^16^ hydrogenase/BOD BFCs. For the *Dv*H-[NiFeSe] bioanode an identical OCV was measured (Fig. 5b). To ensure limiting conditions for the bioanode, an oversized biocathode was used in combination with appropriate glucose concentrations between 1 to 3 mM (same concentration in both compartments was used). Cyclic voltammograms measured with the individual half cells show that the absolute oxidation currents of the bioanodes (Supplementary Fig.11a, c) are indeed smaller than the absolute reduction current at the biocathode (Supplementary Figs. 11b–d) which assures the bioanode being the limiting electrode in all experiments. Even under these harsh conditions the *Dv*MF-[NiFe] bioanode is stable and the corresponding BFC shows a maximum power density of 200 µW cm^-2^ at 0.85 V (referenced to the surface area of the anode). The *Dv*H-[NiFeSe] system shows even higher current densities and a maximum power density of 530 µW cm^-2^ at 0.85 V. Cyclic voltammograms (Supplementary Fig. 11, dashed lines) of the individual half cells measured after the BFC test show pronounced catalytic currents and the signals of the viologen redox couple indicating that no deactivation occurs within the timescale of the experiment. Interestingly, the catalytic currents for the cathodes are much higher after the experiment, most likely due to an accumulation of H_2_O_2_ in the porous electrode structure during the BFC test. Different BFCs prepared with the same enzyme batch show similar values (Supplementary Fig. 12).

The operational stability of the two-compartment BFC was evaluated at a constant load. Figure 5c and d shows the power output over 1 h at 0.8 V for the *Dv*MF-[NiFe] and *Dv*H-[NiFeSe] based BFC, respectively. Only a slight drop of the initial current from 123 µW cm^-2^ (*t* = 10 min) to 113 µW cm^-2^ (*t* = 60 min) for the [NiFe] based system and from 114 µW cm^-2^ (*t* = 10 min) to 100 µW cm^-2^ (*t* = 60 min) for the [NiFeSe] based system was observed. The operational stability is similar to a high current-density hydrogen BFC that is working in a DET regime using a thermostable and O_2_ tolerant hydrgenase^52^.

The long-term stability at a constant load of 0.8 V was evaluated in a two-compartment cell configuration with a *Dv*H-[NiFeSe] based BFC (Supplementary Fig. 15a). The power output reaches 50% of its initial value after ≈ 10 h. After 20 h still 25% of the initial power output could be detected. A closer voltammetric inspection of the individual electrodes (Supplementary Fig. 15b and c) revealed that the turnover currents at the Py_2_Ox/HRP based biocathode even after the addition of further glucose to the solution significantly decreased and are close to the background current. While the currents of the viologen units remain constant before and after the long-term test. The H_2_ oxidation currents decreased by about 30% indicating that parts of the hydrogenase were disintegrated at the bioanode. The results clearly demonstrate that the biocathode is the limiting electrode with respect to durability and that the bioanode shows the envisaged high stability even under operational conditions.

Since the protection system is able to remove significant amounts of detrimental O_2_ at the anode, also a membrane-free single-compartment cell was evaluated using a bioanode based on the highly active *Dv*H-[NiFeSe] hydrogenase (Supplementary Fig. 13). To avoid critical H_2_/O_2_ ratios, the oxygen content in this system was kept constant at 3%, which results in lower currents for the cathode reaction. However, the anode is still the limiting electrode as shown in the cyclic voltammograms of the half cells (Supplementary Fig. 14, red solid lines). The corresponding single-compartment BFC reveals a *P*_max_ of 160 µW cm^−2^ at 0.85 V. The OCV was 1.15 V and thus identical to the two-compartment cell.

Comparison of the cyclic voltammograms of the biocathode under turnover conditions after BFC evaluation show that in the single-compartment configuration the activity of the HRP biocathode was almost completely lost (Supplementary Fig. 14, dashed lines). The bioanode shows a similar current response before and after the BFC test (Supplementary Fig. 14a). The lower power output and lower stability of the one-compartment cell compared to the two-compartment system might be a result of unwanted side reactions that are induced by intermixing all substrates/products in the cell which may lead to a chemical disintegration of especially the biocathode. For the hydrogenase/BOD based systems where no glucose and H_2_O_2_ is present such effects were not observed. This reflects a clear limitation of the system reported here. However, as indicated by voltammograms measured before and after the BFC test, which are almost identical, the bienzymatic protection system in combination with the viologen-modified polymer matrix shows the envisaged full protection of the hydrogenase-based bioanode.

In contrast, when an inactivated catalase was used for the preparation of the double-layer bioanode ([NiFeSe] based system), the evaluation of the fully assembled fuel cell was not possible due to the fast damage of the bioanode with the current dropping to zero within a short time (Supplementary Fig. 16a). Comparison of cyclic voltammograms recorded before and after the BFC test unambiguously shows that not only the hydrogenase itself but also the redox polymer matrix was destroyed due to the local high H_2_O_2_ concentrations during testing. This is evident from the complete loss in H_2_ oxidation currents as well as the redox transition of the polymer-bound viologen moieties after the BFC test (Supplementary Fig. 17a, b). Moreover, also the biocathode took severe damage and its catalytic response was significantly reduced after the BFC evaluation (Supplementary Fig. 17c, d). This deactivation may be related to a voltage drop at the cathode caused by a change of the OCV due to the continuous disintegration of the bioanode leading to the destruction of the biocatalyst by applying extreme potentials. However, the exact nature of this deactivation of the biocathode remains unclear. Most importantly, the developed bioanode with full protection remains active even under harsh conditions (see, e.g., Supplementary Fig. 14a).

## Discussion

The bienzymatic oxidase/catalase second layer immobilized in a redox-silent hydrophilic polymer matrix ensures an efficient protection of the highly O_2_-sensitive *Dv*MF-[NiFe] and *Dv*H-[NiFeSe] hydrogenases. Protection from O_2_ is completely covered by the bienzymatic system and no electrons from the H_2_ oxidation are necessary to be wasted for additional O_2_ reduction at the polymer-bound viologen moieties. Thus, the bioanode provides the envisaged constant current response even in the presence of O_2_. Moreover, using Py_2_Ox instead of GOx in the O_2_ removal system provides a stable pH value even during long-term runs. However, the higher stability is counterbalanced by a lower protection efficiency of the Py_2_Ox system (3% O_2_) compared to the GOx-based system (5% O_2_).

Even though O_2_ protection is decoupled from the polymer/hydrogenase reaction layer, the viologen-modified redox polymer is important not only as a fast electron-transfer redox mediator for the entrapped hydrogenase, but also since its low potential furthermore protects the hydrogenase from high-potential deactivation and ensures removal of O_2_ traces that may break through the bienzymatic O_2_ removal layer. Evidently, by incorporation of sensitive hydrogenases in a viologen-modified polymer layer in combination with a top layer comprising the bienzymatic O_2_ removal system the proposed bioanode configuration ensures a complete protection of the hydrogenase even under continuous operation.

The low potential of the viologen-modified polymer allows for H_2_ oxidation to occur at low overpotentials, which in combination with a biocathode based on HRP that is wired via the high potential iron-oxo complex compound I ensures a benchmark OCV of around 1.15 V for a polymer-based H_2_/glucose(H_2_O_2_) BFC.

In the presence of H_2_ and glucose, which simultaneously acts as reactant for the O_2_ removal layer at the bioanode and as enzyme substrate for the formation of the oxidant H_2_O_2_ at the biocathode, a maximum power density of about 0.5 mW cm^-2^ at a remarkably high cell voltage of ≈ 0.85 V was achieved when the highly active [NiFeSe] hydrogenase from *Desulfovibrio vulgaris* Hildenborough was used. The *Dv*H-[NiFeSe] containing BFC reveals a performance that outperforms a previously reported polymer/hydrogenase systems using a BOD based biocathode^15^. In contrast, the cell is inferior to previously described H_2_/O_2_ hydrogenase-based BFCs operated in a DET mode^6–8^, which are able to reach power densities of up to 8.4 mW cm^-2^ in a gas breathing configuration^7^. However, these systems lack active protection against O_2._

In conclusion, we developed a polymer double-layer air-stable hydrogenase bioanode that was successfully incorporated into a H_2_/glucose(H_2_O_2_) BFC with an extraordinary high OCV that sets a benchmark for redox polymer-based BFCs. The proposed bienzymatic-protection system may be transposed to other O_2_-sensitive biocatalysts or artificial catalysts. Moreover, the integration of additional or other enzymes into the protection layer may allow the establishment of new shielding systems that are able to remove not only O_2_ but also other detrimental interferences that may attack sensitive catalysts of any type and nature.

## Methods

### Chemicals and materials

All chemicals were purchased from Sigma-Aldrich, Alfa-Aesar, Acros Organics, VWR, Merck, J.T. Baker or Deutero and were used as received except otherwise noted. All chemicals were of reagent or higher grade. Gases used for the preparation of the CMF/CNT modified carbon cloth revealed a purity of N6.0. All aqueous solutions were prepared with Milli-Q water from a water purification system (Millipore).

### Polymers

The synthesis and characterization of the viologen-modified polymer P(N_3_MA-BA-GMA)-vio (Fig. 1) was described elsewhere^17^. The actual composition of the polymer backbone was *x* = 71 mol%, *y* = 20 mol%, *z* = 9 mol% (determined by NMR spectroscopy)^17^. The synthesis of the hydrophilic and redox-silent polymer P(SS-GMA-BA) (Fig. 1) was described in ref. ^49^, the nominal concentration of the individual co-monomers was *k* = 50 mol%, *l* = 30 mol%, and *m* = 20 mol%. Note that due to overlapping signals in the NMR spectrum of P(SS-GMA-BA) the actual composition cannot be determined via the integral ratios. However, all characteristic signals could be identified, and their chemical shifts are consistent with the proposed molecular structure^49^. For electrode modifications the polymers were dissolved in water with concentrations of 8 mg mL^−1^ for P(N_3_MA-BA-GMA)-vio and 60 mg mL^−1^ for P(SS-GMA-BA). The polymer stock solutions were stored at room temperature.

### Enzymes

Glucose oxidase (GOx) from *Aspergillus niger* (Type X-S, lyophilized powder, 100,000-250,000 U g^−1^ solid), catalase (CAT) from bovine liver (lyophilized powder, 2,000-5,000 U mg^−1^ protein), pyranose oxidase (Py_2_Ox) from *Coriolus* sp. (recombinant, expressed in *E. coli*, ≥ 2.7 U mg^−1^ solid) and HRP (Type VI-A, lyophilized powder, salt free, 325 U mg^−1^) were purchased from Sigma-Aldrich and stored at −20 °C. Stock solutions with enzyme concentrations of 10 mg mL^−1^ were prepared in phosphate buffer (0.1 M, pH 7.4) and stored at 4 °C.

The [NiFe] hydrogenase from *Desulfovibrio vulgaris* Miyazaki F (*Dv*MF-[NiFe]) was isolated and purified according to ref. ^53,54^. It was stored in MES buffer at pH 6.8 at −20 °C with a concentration of 200 µM. The H_2_ production activity of this type of hydrogenase was determined to be 610 U mg^−1,^^54^. However, the activities of the individual batches varied and electrodes showed different activities toward the oxidation of H_2_ ( ± 50%, with respect to the average value). The recombinant form of the [NiFeSe] hydrogenase from *Desulfovibrio vulgaris* Hildenborough (*Dv*H-[NiFeSe]) was isolated and purified as described previously in ref. ^48^. The activity for H_2_ formation was estimated to be 4000–5700 U mg^−1^, depending on the batch with variations of up to 320 U mg^−1^ for each single batch. The activity of modified electrodes varied by ±90% (with respect to the average value). The enzyme was stored at −80 °C in 20 mM Tris-HCl at pH 7.6 with a concentration of 14–15 µg µL^−1^ (159–170 µM). Note that rather high concentrations of the enzymes were used to achieve high biocatalyst loading on the electrode surface while keeping the volumes use the drop cast process rather small to minimize drying time and facilitate the modification of the 3 mm electrodes.

Although, the activity of the individual electrodes toward H_2_ oxidation varied for different enzyme batches which lead to a variation in the absolute current response, all electrodes showed the same trend under different conditions thus allowing a qualitative comparison of the individual systems.

### Electrochemical experiments

All voltammetric and chronoamperometric experiments were conducted in a standard three electrode gas-tight electrochemical cell under Ar atmosphere or Ar/H_2_/O_2_ mixtures at room temperature using a Reference 600 (Gamry Instruments), an Autolab PGSTAT12 (Metrohm-Autolab) or an Autolab FRA 2 Type III (Metrohm-Autolab) potentiostat. The counter electrode was a Pt wire. As reference electrode a Ag/AgCl/3 M KCl system was used. All potentials are rescaled with respect to the standard hydrogen electrode (SHE) which is +210 mV more negative than the Ag/AgCl/3 M KCl system. For the fabrication of the hydrogenase-bioanodes glassy carbon electrodes with a nominal diameter of 3 mm and thus a geometrical surface area of 0.07 cm^2^ were used. Phosphate buffer (PB, 100 mM, pH 7.4) served as working electrolyte for all measurements. For measurements with different Ar/H_2_/O_2_ ratios three separated mass flow controllers were used to control the gas flow of the individual gases. The mass flow controllers were directly connected to the cell via a single gas-inlet. The gases were pre-mixed and then purged through the electrolyte solution and no additional stirring or rotation of the electrode was applied. Prior to each experiment, the glassy carbon working electrodes were subsequently polished using several alumina/water suspensions with decrease particle grain size (going from 1 µm, via 0.3 µm to 0.05 µm), each with ≈ 1 min. After each polishing step the electrodes were thoroughly rinsed with water. After final polishing step the electrodes were again washed with water and dried in an argon stream to remove any dust particles.

BFC tests were either conducted in a one- or two-compartment cell by using a hydrogenase bioanode and a GOx/HRP or Py_2_Ox/HRP biocathode. As separator in the two-compartment cell a Nafion membrane was used. Both compartments revealed a gas outlet to avoid extensive mixing of H_2_ and O_2_. H_2_ and O_2_ were bubbled through the corresponding compartment. For the one-compartment cell, a gas mixture of 90% H_2_/5% O_2_/5% Ar was used. Power curves were obtained by stepped potential chronoamperometric experiments. This technique minimizes contributions from background currents. Steady state currents were used to calculate the power value of the corresponding BFCs. To ensure anode limiting conditions, the oxygen flow in the cathodic compartment of the two-compartment cell was adjusted to achieve absolute currents that were significantly higher than those measured at the anode. Since for all experiments the bioanode acted as the limiting electrode, a surface area of 0.07 cm^2^ was used to calculate current and power densities.

### Fabrication of CNT/CMF-modified carbon cloth electrodes

For fabrication of the biocathodes carbon cloth modified with CMFs which were decorated with CNTs were used. These high surface area electrodes were prepared according to protocols described in refs. ^37,38^. For the growth of the CMFs, first an iron-based catalyst was deposited onto the base electrode material in an electrochemical process. The catalyst was deposited from an aqueous solution containing FeSO_4_·7H_2_O (0.50 M) and MgSO_4_·7H_2_O (0.58 M) by applying a multi-potential pulse sequence with *n*(0 V/10 s; −0.6 V/2 s) (with *n* = 20, both potentials vs. Ag/AgCl/3 M KCl). The carbon cloth electrodes were pre-wetted with ethanol and water and then directly immersed into the electrolyte solution containing the iron precursor. As counter electrode a Pt gauze and as reference electrode a Ag/AgCl/3 M KCl system was used. The same procedure was used for the deposition of the iron catalyst on the CMFs for the growth of the CNTs. After CMF/CNT deposition the electrodes were thoroughly washed with water.

For growth of CMFs the iron catalyst decorated electrode material was placed in a quartz crucible and mounted in a quartz tube (100 cm length with an inner diameter of 3 cm). The tube was placed in a triple-zone tube furnace (Carbolite) and connected to a computer-controlled mass flow controller system. The sample was heated to 850 °C with an increase of 20 °C min^−1^ and then to 1150 °C with 3 °C min^−1^ in a H_2_/CH_4_ mixture (2.3:1). After 20 min at 1150 °C the temperature was lowered to 950 °C under pure H_2_ atmosphere. Then, the sample was allowed to cool down to room temperature under 100% He atmosphere.

For CNT growth on the CMFs, the same setup was used. First the temperature was raised to 700 °C (10 °C min^−1^) under a H_2_/He atmosphere (1:1). The temperature was increased to 760 °C (10 °C min^−1^) and kept constant for 20 min in a gas mixture if H_2_/C_2_H_4_ (2.3:1). The tube was flushed with 100% H_2_ for additional 30 min and finally the sample cooled down to room temperature in He atmosphere.

The electrodes were characterized by means of scanning electron microscopy. Supplementary Fig. 8a shows the bare carbon cloth. Supplementary Fig. 8b depicts the CMF modified carbon cloth with a diameter of the CMFs of ≈ 0.5 µM randomly distributed on the carbon cloth fibers. The CNT decorated material reveals a porous structure as shown in Supplementary Fig. 8c.

### Electrode modification

Bioanodes: for the preparation of the hydrogenase-bioanodes glassy carbon electrodes (diameter = 3 mm) were first modified with a hydrogenase/P(N_3_MA-BA-GMA)-vio layer by pre-mixing an aqueous solution of the polymer (8 mg mL^−1^ in water, 3 µL), the enzyme solution (*Dv*MF-[NiFe]: 200 µM in MES pH 6.8, 0.5 µL; *Dv*H-[NiFeSe]: 159–170 µM in Tris-HCL at pH 7.8, 0.5 µL) and Tris-HCl-buffer (0.1 M, pH 8.6, 3 µL). Electrodes were then modified by drop casting (1.3 µL polymer/enzyme mixture) and dried at 4 °C for ≈4 h. The enzymatic-protection layer comprising GOx and CAT embedded in P(SS-GMA-BA) was added by drop coating on top of the dried polymer/hydrogenase layer. For this, the polymer P(SS-GMA-BA) (60 mg mL^−1^ in water, 10 µL), GOx (10 mg mL^−1^ in 0.1 M PB, pH 7.4, 10 µL) or Py_2_Ox (10 mg mL^−1^ in 0.1 M PB, pH 7.4, 10 µL), CAT (10 mg mL^−1^ in 0.1 M PB, pH 7.4, 10 µL) and the bifunctional crosslinker 2,2′-(ethylenedioxy)diethylamine (1:37 in water, 1 µL) was pre-mixed and drop coated (31 µL) onto the modified electrode in a way that the underlying reaction layer was homogeneously covered. Finally, the modified electrodes were dried under ambient conditions over night.

For measurements with only the hydrogenase/polymer or GOx/CAT/polymer bioanodes, respectively, the electrodes were prepared using the same polymer/enzyme/crosslinker ratios.

Biocathodes: Oxidase/HRP-modified carbon cloth electrodes were prepared in a sequential process starting with the modification of the CNT/CMF decorated carbon cloth (geometrical surface area = 1 cm^2^) with pyrene butyric acid (PBA). For this, the electrode was immersed for 1 h into dimethylformamide containing PBA at a concentration of 20 mg mL^−1^. The PBA modified electrode was thoroughly rinsed with dimethylformamide and water. In a second step the surface was modified with HRP (10 mg mL^−1^, 20 µL) by drop casting. After drying, the second enzyme layer, i.e. GOx or Py_2_Ox, was added (10 mg mL^−1^, 20 µL). The modified electrodes were dried and rinsed with water to remove any loosely bound enzyme.

## Electronic supplementary material

Supllementary Information

Peer Review File

## Data Availability

The data that support the findings of this study are available from the corresponding author upon reasonable request.

## References

[1] Lubitz W, Ogata H, Rüdiger O, Reijerse E (2014). Hydrogenases. Chem. Rev..

[2] Jones, A. K., Sillery, E., Albracht, S. P. J. & Armstrong, F. A. Direct comparison of the electrocatalytic oxidation of hydrogen by an enzyme and a platinum. *Chem. Commun*. **8**, 866–867 (2002). 10.1039/b201337a 12123018

[3] Karyakin AA (2007). The limiting performance characteristics in bioelectrocatalysis of hydrogenase enzymes. Angew. Chem. Int. Ed..

[4] Varfolomeev SD, Yaropolov AI, Berezin IV, Tarasevich MR, Bogdanovskaya VA (1977). Bioelectrocatalysis. Hydrogenase as catalyst of electrochemical hydrogen ionization. Bioelectrochem. Bioenerg..

[5] Tsujimura S, Fujita M, Tatsumi H, Kano K, Ikeda T (2001). Bioelectrocatalysis-based dihydrogen/dioxygen fuel cell operating at physiological pH. Phys. Chem. Chem. Phys..

[6] Mazurenko I (2017). Impact of substrate diffusion and enzyme distribution in 3D-porous electrodes. A combined electrochemical and modelling study of a thermostable H_2_ /O_2_ enzymatic fuel cell. Energy Environ. Sci..

[7] So K (2016). Direct electron transfer-type dual gas diffusion H_2_/O_2_ biofuel cells. J. Mater. Chem. A.

[8] Mazurenko I, Wang X, Poulpiquet Ade, Lojou E (2017). H_2_/O_2_ enzymatic fuel cells. From proof-of-concept to powerful devices. Sustain. Energy Fuels.

[9] Mazurenko I, Poulpiquet Ade, Lojou E (2017). Recent developments in high surface area bioelectrodes for enzymatic fuel cells. Curr. Opin. Electrochem..

[10] Cosnier S, J. Gross A, Le Goff A, Holzinger M (2016). Recent advances on enzymatic glucose/oxygen and hydrogen/oxygen biofuel cells. Achievements and limitations. J. Power Sources.

[11] Falk M, Blum Z, Shleev S (2012). Direct electron transfer based enzymatic fuel cells. Electrochim. Acta.

[12] Xu L, Armstrong FA (2015). Pushing the limits for enzyme-based membrane-less hydrogen fuel cells – achieving useful power and stability. RSC Adv..

[13] Radu V, Frielingsdorf S, Evans SD, Lenz O, Jeuken LJC (2014). Enhanced oxygen-tolerance of the full heterotrimeric membrane-bound NiFe-hydrogenase of Ralstonia eutropha. J. Am. Chem. Soc..

[14] Morozov S (2006). Tolerance to oxygen of hydrogen enzyme electrodes. Electrochem. Commun..

[15] Plumeré N (2014). A redox hydrogel protects hydrogenase from high-potential deactivation and oxygen damage. Nat. Chem..

[16] Oughli AA (2015). A redox hydrogel protects the O_2_-sensitive FeFe-hydrogenase from Chlamydomonas reinhardtii from oxidative damage. Angew. Chem. Int. Ed..

[17] Ruff A (2017). Protection and reactivation of the [NiFeSe] hydrogenase from *Desulfovibrio vulgaris Hildenborough* under oxidative conditions. ACS Energy Lett..

[18] Fourmond V (2015). Mechanism of protection of catalysts supported in redox hydrogel films. J. Am. Chem. Soc..

[19] Plumeré N, Henig J, Campbell WH (2012). Enzyme-catalyzed O_2_ removal system for electrochemical analysis under ambient air. Application in an amperometric nitrate biosensor. Anal. Chem..

[20] Benesch RE, Benesch R (1953). Enzymatic removal of oxygen for polarography and related methods. Science.

[21] Meyer AS, Isaksen A (1995). Application of enzymes as food antioxidants. Trends Food Sci. Technol..

[22] Plumeré N (2013). Interferences from oxygen reduction reactions in bioelectroanalytical measurements. The case study of nitrate and nitrite biosensors. Anal. Bioanal. Chem..

[23] Lopez F, Zerria S, Ruff A, Schuhmann W (2018). An O_2_ tolerant polymer/glucose oxidase based bioanode as basis for a self-powered glucose sensor. Electroanalysis.

[24] Mano N, Mao F, Heller A (2002). A miniature biofuel cell operating in a physiological buffer. J. Am. Chem. Soc..

[25] Le Goff A, Holzinger M, Cosnier S (2015). Recent progress in oxygen-reducing laccase biocathodes for enzymatic biofuel cells. Cell. Mol. Life Sci..

[26] Barton SC, Kim HH, Binyamin G, Zhang Y, Heller A (2001). Electroreduction of O_2_ to Water on the “Wired” Laccase Cathode. J. Phys. Chem. B.

[27] Barton SC, Pickard M, Vazquez-Duhalt R, Heller A (2002). Electroreduction of O_2_ to water at 0.6V (SHE) at pH 7 on the ‘wired’ Pleurotus ostreatus laccase cathode. Biosens. Bioelectron..

[28] Wang X (2012). Mediatorless sugar/oxygen enzymatic fuel cells based on gold nanoparticle-modified electrodes. Biosens. Bioelectron..

[29] Salaj-Kosla U, Pöller S, Schuhmann W, Shleev S, Magner E (2013). Direct electron transfer of Trametes hirsuta laccase adsorbed at unmodified nanoporous gold electrodes. Bioelectrochemistry.

[30] Hirose J (1998). Anions binding to bilirubin oxidase from Trachyderma tsunodae K-2593. Inorg. Chim. Acta.

[31] Mano N, Poulpiquet Ade (2017). O_2_ Reduction in enzymatic biofuel cells. Chem. Rev..

[32] Milton RD, Giroud F, Thumser AE, Minteer SD, Slade RCT (2014). Bilirubin oxidase bioelectrocatalytic cathodes. The impact of hydrogen peroxide. Chem. Commun..

[33] Scodeller P (2010). Layer-by-layer self-assembled osmium polymer-mediated laccase oxygen cathodes for biofuel cells: the role of hydrogen peroxide. J. Am. Chem. Soc..

[34] Milton RD, Giroud F, Thumser AE, Minteer SD, Slade RCT (2013). Hydrogen peroxide produced by glucose oxidase affects the performance of laccase cathodes in glucose/oxygen fuel cells. FAD-dependent glucose dehydrogenase as a replacement. PhysChemChemPhys.

[35] Rich PR, Iwaki M (2007). A comparison of catalytic site intermediates of cytochrome c oxidase and peroxidases. Biochemistry.

[36] Yamazaki, I., Tamura, M. & Nakajima, R. Horseradish peroxidase C. *Mol. Cell Biochem*. **40**, 143–153(1981). 10.1007/BF00224608 7322114

[37] Jia W (2012). Glucose oxidase/horseradish peroxidase co-immobilized at a CNT-modified graphite electrode. Towards potentially implantable biocathodes. Eur. J. Chem..

[38] Jia W (2010). Towards a high potential biocathode based on direct bioelectrochemistry between horseradish peroxidase and hierarchically structured carbon nanotubes. PhysChemChemPhys.

[39] Agnès C, Reuillard B, Le Goff A, Holzinger M, Cosnier S (2013). A double-walled carbon nanotube-based glucose/H_2_O_2_ biofuel cell operating under physiological conditions. Electrochem. Commun..

[40] Reuillard B, Le Goff A, Holzinger M, Cosnier S (2014). Non-covalent functionalization of carbon nanotubes with boronic acids for the wiring of glycosylated redox enzymes in oxygen-reducing biocathodes. J. Mater. Chem. B.

[41] Krikstolaityte V (2013). Biofuel cell based on anode and cathode modified by glucose oxidase. Electroanalysis.

[42] Elouarzaki K (2015). Freestanding HRP–GO_*x*_ redox buckypaper as an oxygen-reducing biocathode for biofuel cell applications. Energy Environ. Sci..

[43] Lidén H, Volc J, Marko-Varga G, Lo Gorton (1998). Pyranose oxidase modified carbon paste electrodes for monosaccharide determination. Electroanalysis.

[44] Jönsson G, Gorton L (1989). An electrochemical sensor for hydrogen peroxide based on peroxidase adsorbed on a spectrographic graphite electrode. Electroanalysis.

[45] Johansson K, Jönsson-Pettersson G, Gorton L, Marko-Varga G, Csöregi E (1993). A reagentless amperometric biosensor for alcohol detection in column liquid chromatography based on co-immobilized peroxidase and alcohol oxidase in carbon paste. J. Biotechnol..

[46] Ruff A, Pinyou P, Nolten M, Conzuelo F, Schuhmann W (2017). A self-powered ethanol biosensor. ChemElectroChem.

[47] Pandelia ME, Ogata H, Lubitz W (2010). Intermediates in the catalytic cycle of NiFe hydrogenase. Functional spectroscopy of the active site. Chemphyschem.

[48] Marques MC (2017). The direct role of selenocysteine in NiFeSe hydrogenase maturation and catalysis. Nat. Chem. Biol..

[49] Teanphonkrang S (2018). Tuned amperometric detection of reduced β-nicotinamide adenine dinucleotide by allosteric modulation of the reductase component of the p-hydroxyphenylacetate hydroxylase immobilized within a redox polymer. Anal. Chem..

[50] Lopez F, Ma S, Ludwig R, Schuhmann W, Ruff A (2017). A polymer multilayer based amperometric biosensor for the detection of lactose in the presence of high concentrations of glucose. Electroanalysis.

[51] Bartlett PN, Pratt K (1995). Theoretical treatment of diffusion and kinetics in amperometric immobilized enzyme electrodes Part I. Redox mediator entrapped within the film. J. Electroanal. Chem..

[52] Lalaoui N (2015). A membraneless air-breathing hydrogen biofuel cell based on direct wiring of thermostable enzymes on carbon nanotube electrodes. Chem. Commun..

[53] Fichtner C, Laurich C, Bothe E, Lubitz W (2006). Spectroelectrochemical characterization of the NiFe hydrogenase of *Desulfovibrio vulgaris Miyazaki F*. Biochemistry.

[54] Yagi T (1976). Properties of purified hydrogenase from the particulate fraction of desulfovibrio vulgaris, Miyazaki1. J. Biochem..

