# Supplementary material for: A fully protected hydrogenase/polymer-based bioanode for high-performance hydrogen/glucose biofuel cells
Source: Nat Commun. 2018 Sep 10;9:3675. doi: 10.1038/s41467-018-06106-3 (PMC6131248; doi:10.1038/s41467-018-06106-3)
Supplement: Supplementary file 1 — Supllementary Information [file 41467_2018_6106_MOESM1_ESM.pdf]

Supplementary Information

A fully protected hydrogenase/polymer based bioanode for hydrogen/glucose biofuel cells

Ruff et al.

Supplementary Figures

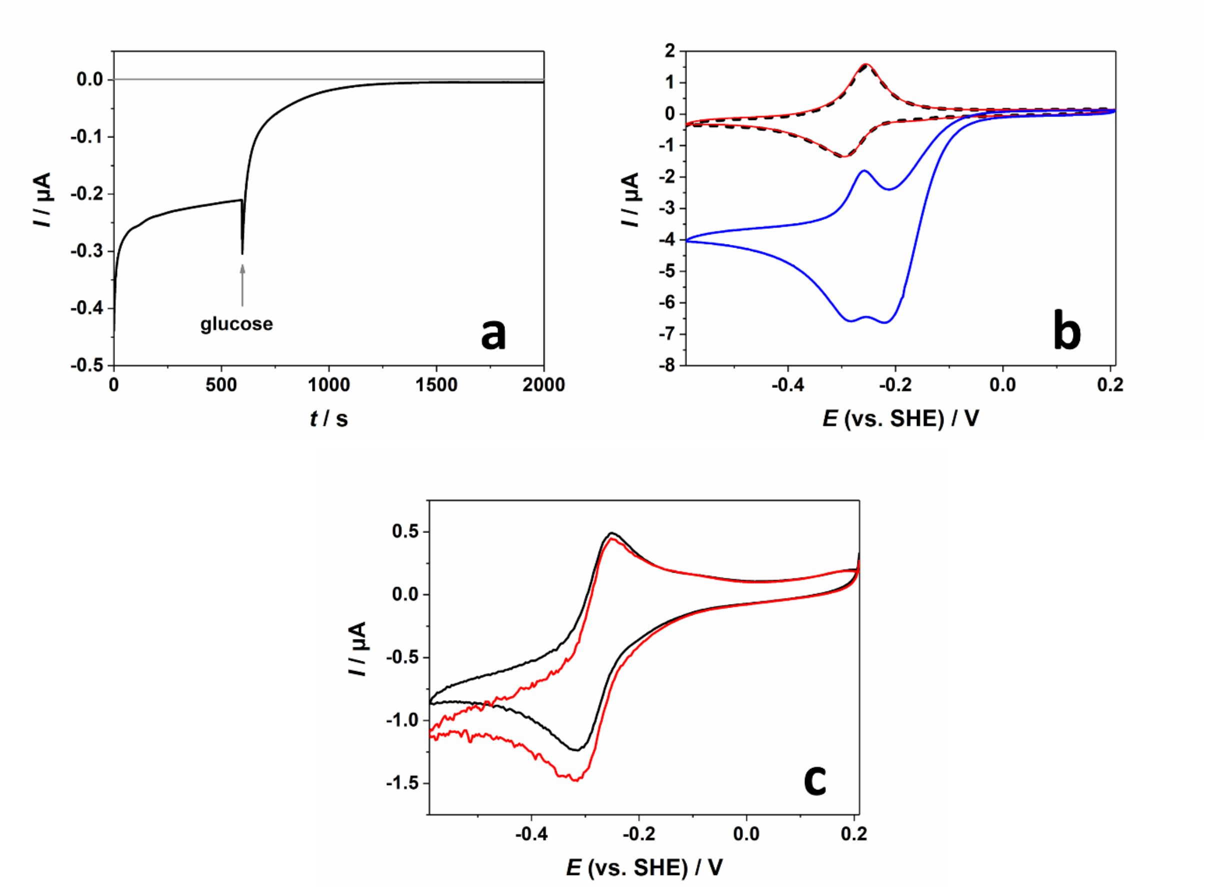

Supplementary Figure 1: Characteristics of the O_2 removal system based on P(SS-GMA-BA)/GOx/CAT. **a**: Chronoamperometric experiment with a P(SS-GMA-BA)/GOx/CAT (2:1:1 wt%) modified Pt-electrode at an applied potential of +10 mV vs. SHE under air in quiescent solution. After the addition of glucose (50 mM) at t = 595 s, the current drops to zero since the bi-enzymatic system starts to remove O_2 in front of the electrode surface. **b**: Cyclic voltammograms in quiescent solution of a P(N_3MA-BA-GMA)-vio//P(SS-GMA-BA)/GOx/CAT multilayer film deposited on a GC electrode under argon (black dashed line), under air without glucose in solution (blue line), and under air with glucose in solution (red line). **c**: Cyclic voltammograms of a P(N_3MA-BA-GMA)-vio/*Dv*MF-[NiFe]//P(SS-GMA-BA)/GOx/CAT double-layer film on GC while purging of argon (black line) or a mixture of 5 % O_2 and 95 % Ar (red line) through the electrolyte. Working electrolyte for all measurements was 0.1 M PB (pH 7.4). Scan rate: 10 mV s^{-1}.

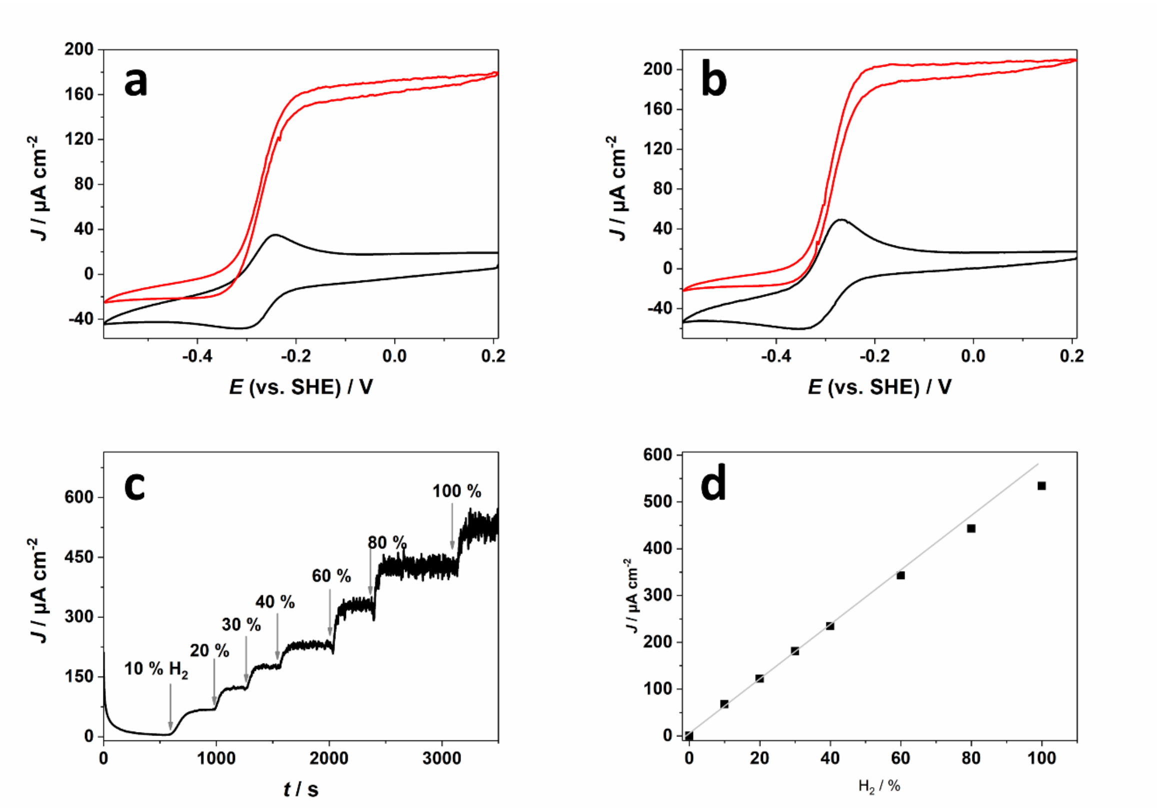

Supplementary Figure 2: Voltammetric (**a** and **b**) and amperometric characterization (**C**) of hydrogenase bioanodes in 0.1 M PB, pH 7.4. **a** and **b**: Cyclic voltammograms of a P(N_3MA-BA-GMA)-vio/*Dv*H-[NiFeSe]//P(SS-GMA-BA)/GOx/CAT (**a**) and P(N_3MA-BA-GMA)-vio/*Dv*MF-[NiFe] (**b**) modified electrode recorded with 10 mV s^{-1} under 100 % argon (black trace) and turnover conditions (100 % H_2), gases were purged through the electrolyte, the same hydrogenase batch was used for both experiments; **c** and **d**: Dependence of the current density *J* on the H_2 concentration in the gas feed determined for a P(N_3MA-BA-GMA)-vio/*Dv*H-[NiFeSe]//P(SS-GMA-BA)/GOx/CAT modified glassy carbon electrode. For the double-layer system, *J* is clearly limited by H_2 mass transport. **c**: plot of *J* vs. *t* and **d**: plot of *J* vs. H_2 content in the gas feed in % showing that a linear increase of *J* was observed for H_2 levels below ≈40 %; chronoamperometric measurements were performed at an applied potential of +160 mV vs. SHE.

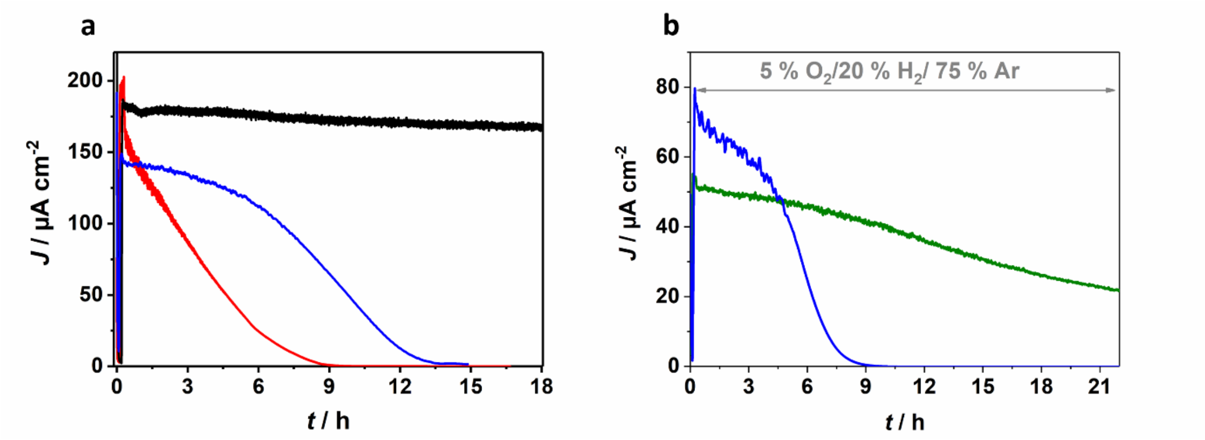

Supplementary Figure 3: Chronoamperometric long-term stability tests of double-layer bioanodes in 0.1 M PB, pH 7.4 at an applied potential of +160 mV vs. SHE under turnover conditions and in the presence of O_2. **a**: black trace: P(N_3MA-BA-GMA)-vio/*Dv*MF-[NiFe]//P(SS-GMA-BA)/GOx/CAT system with 20 % H_2/80 % Ar bubbling through the cell and in the absence of glucose; red trace: single P(N_3MA-BA-GMA)-vio/*Dv*MF-[NiFe] layer, 5 % O_2/20 % H_2/ 75 % Ar; blue trace: P(N_3MA-BA-GMA)-vio/*Dv*MF-[NiFe]//P(SS-GMA-BA)/GOx/CAT system with 5 % O_2/20 % H_2/ 75 % Ar bubbling through the cell and 100 mM glucose; the same enzyme batch was used for all three experiments. **b**: Comparison between the protection systems based on GOx (P(N_3MA-BA-GMA)-vio/*Dv*MF-[NiFe]//P(SS-GMA-BA)/GOx/CAT, green line) and Py_2Ox (P(N_3MA-BA-GMA)-vio/*Dv*MF-[NiFe]//P(SS-GMA-BA)/Py_2Ox/CAT, blue line) with 5 % O_2/20 % H_2/ 75 % Ar bubbling through the electrolyte and with 50 mM glucose in solution; the same enzyme batch was used for both measurements. Absolute current densities are shown, for normalized values see Figure 3 in the main text. Note that for the experiments depicted in **a** and **b** respectively, two different hydrogenase batches were used which explains the differences in absolute currents in **a** and **b**.

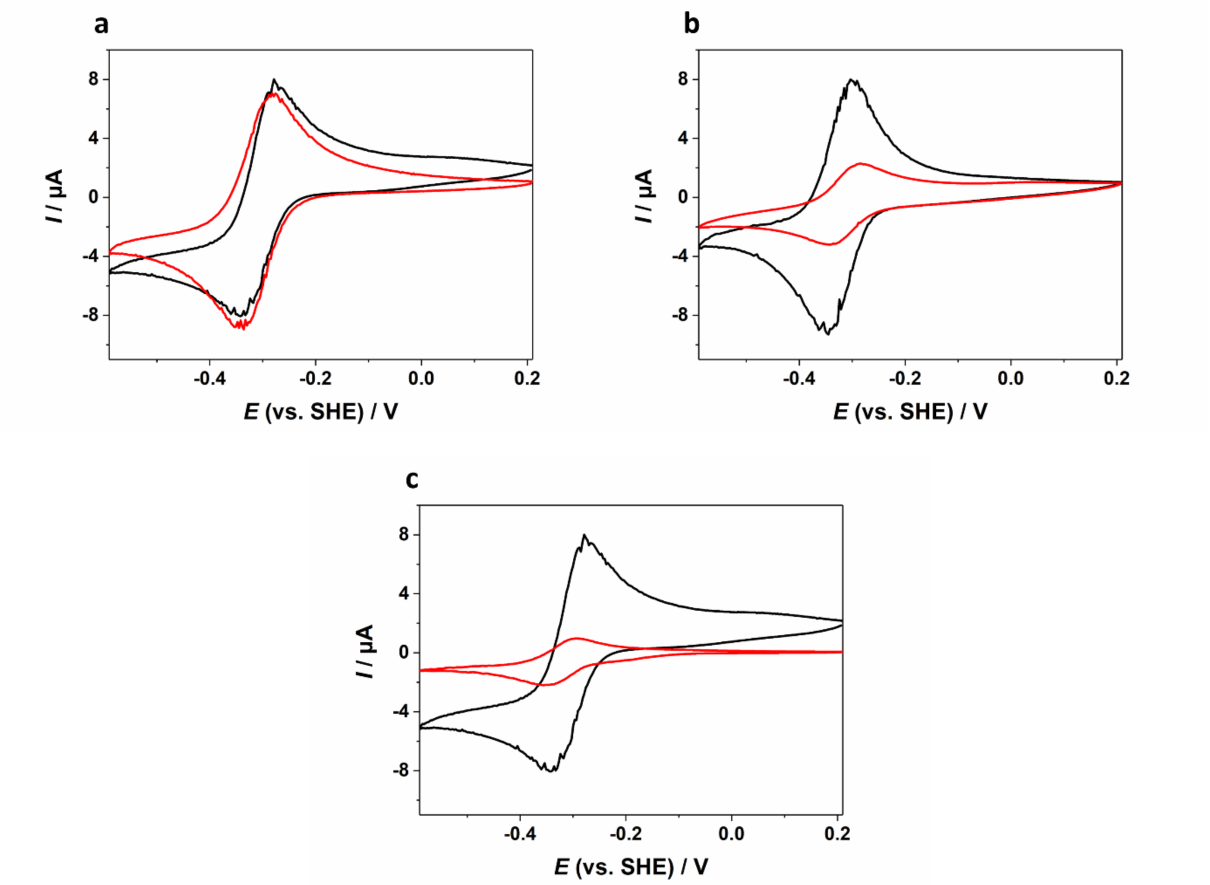

Supplementary Figure 4: Cyclic voltammograms of P(N_3MA-BA-GMA)-vio/*Dv*MF-[NiFe]//P(SS-GMA-BA)/GOx/CAT coated glassy carbon electrodes in 0.1 PB, pH 7.4 under argon with a scan rate of 10 mV s^{-1} before (black lines) and after (red lines) the long-term measurements as shown in Figure 3A in the main text. Conditions for long-term measurements: **a**: 20 % H_2/80 % Ar; **b**: 20 % H_2/75 % Ar/5 % O_2, the electrode was only modified with a P(N_3MA-BA-GMA)-vio/*Dv*MF-[NiFe] layer, the protection layer was absent; **c**: 20 % H_2/75 % Ar/5 % O_2 and 100 mM glucose in solution.

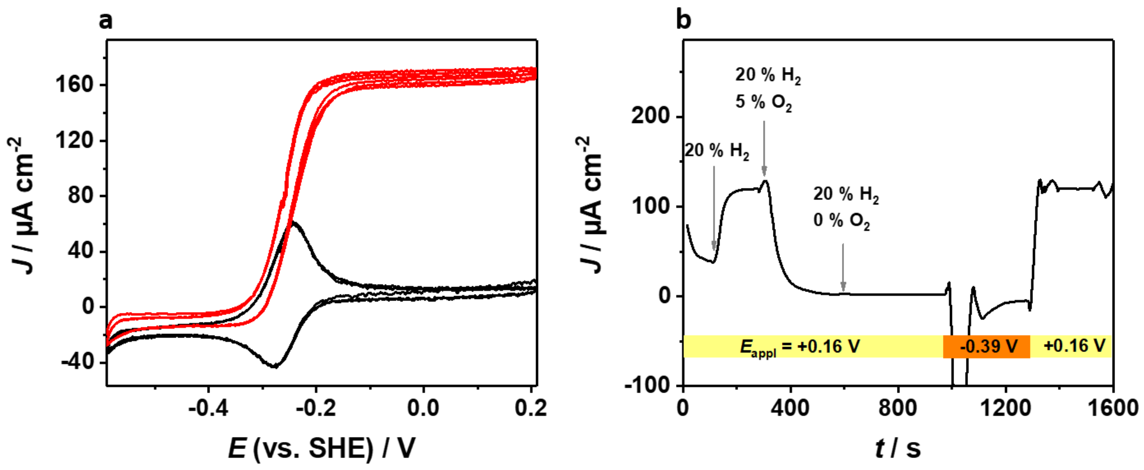

Supplementary Figure 5: Reactivation of *Dv*MF-[NiFe] that was deactivated by O_2 in electrochemically deposited P(N_3MA-BA-GMA)-vio/*Dv*MF-[NiFe] thin films. For the deposition a pulse sequence of n (+1.71 V/0.2 s; +0.21V/2 s) with n = 10 (E vs. SHE) and an electrochemically activated crosslinker was used according to procedures described in ref. [1]. Experiments were conducted in 0.1 M phosphate buffer (pH 7.4). **a**: Cyclic voltammograms of the P(N_3MA-BA-GMA)-vio/*Dv*MF-[NiFe] thin film under argon (black line) and H_2 atmosphere (red line), three consecutive scans are shown for each case; scan rate = 10 mV s^{-1}. The voltammograms show that even in thin films a mediated electron transfer is present: the half wave potential of the catalytic waves matches the redox potential of the viologen-based mediator; thus, the hydrogenases are in electrical contact with the viologen units and electrons can be exchanged. **b**: Chronoamperometric experiment with different gas feeds and different applied potentials (E_{appl} vs. SHE). The argon content in the gas feed was adjusted to achieve the desired composition of the H_2/O_2/Ar mixture. In thin films, the oxygen front is immediately reaching the reaction layer and deactivates the biocatalyst ($t > 350$ s). After the O_2 feed was stopped (t = 600 s), the current remains at the background value, indicating that the enzyme is fully deactivated by O_2. When the polymer is reduced at an applied potential of -0.39 V (t = 900-1300 s), the redox mediator can reactivate the inactivated enzyme by reduction and the oxidation current was restored ($t > 1300$ s). This effect was first observed for the *Dv*H-[NiFeSe] hydrogenase and the reader is referred to ref. [1] for a more detailed description of this process and thin film formation. For the *Dv*MF-[NiFe] this effect was not described yet, and this is the first time that reactivation was shown for this hydrogenase.

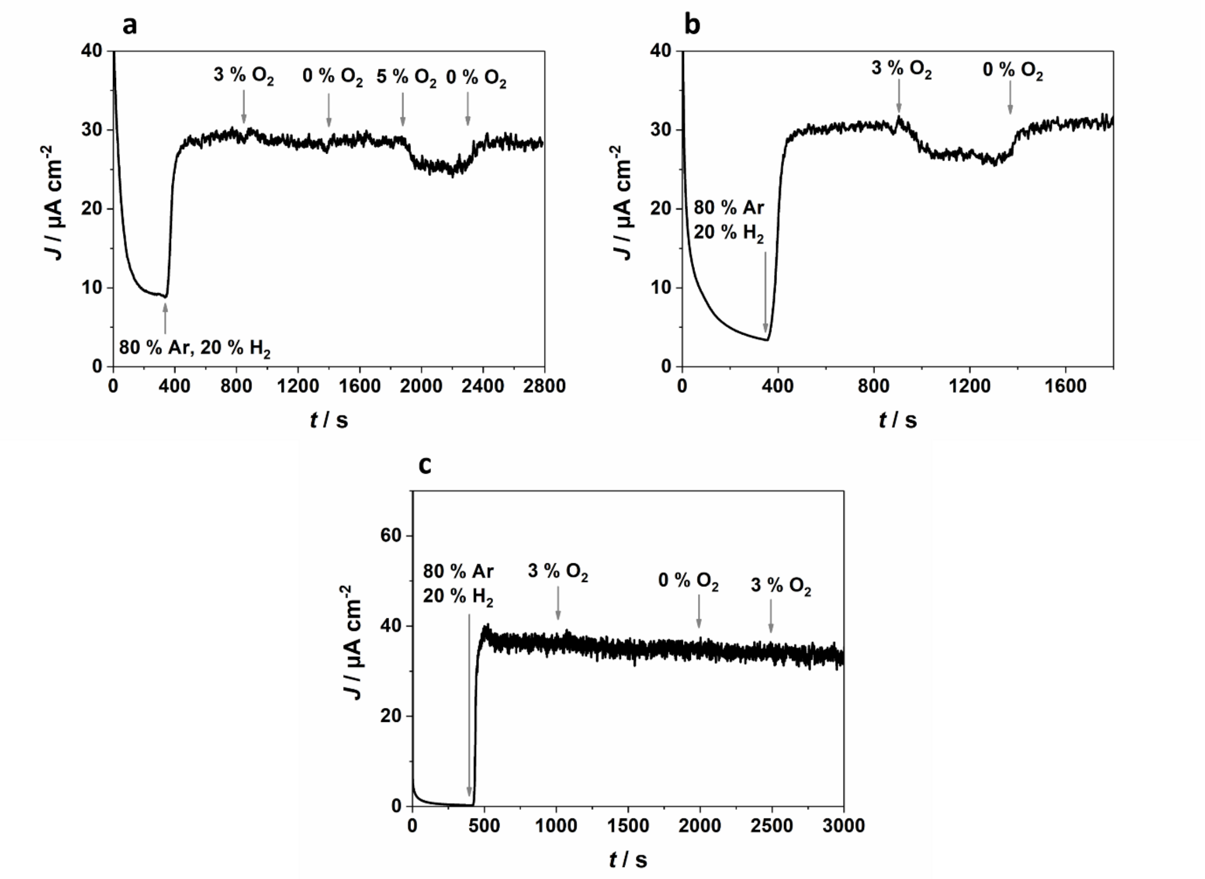

Supplementary Figure 6: Characterization of the P(SS-GMA-BA)/Py_2Ox/CAT protection system by means of chronoamperometry in 0.1 M PB, pH 7.4 at an applied potential of +160 mV vs. SHE. **a**: Current density for a P(N_3MA-BA-GMA)-vio/*Dv*MF-[NiFe]//P(SS-GMA-BA)/Py_2Ox/CAT double-layer system in the presence of different O_2 concentrations in the gas feed and with 50 mM glucose in solution. **b**: Current density for a P(N_3MA-BA-GMA)-vio/*Dv*MF-[NiFe]//P(SS-GMA-BA)/Py_2Ox/CAT double-layer system in absence of glucose and with varying O_2 content in the gas feed. **c**: Current density for a P(N_3MA-BA-GMA)-vio/*Dv*MF-[NiFe]//P(SS-GMA-BA)/Py_2Ox/CAT double-layer system in presence of 1 mM glucose and with alternatingly switching from 0 to 3 % O_2. The Py_2Ox based protection system provides a full protection for O_2 levels of up to 3 % even at low glucose concentration of only 1 mM.

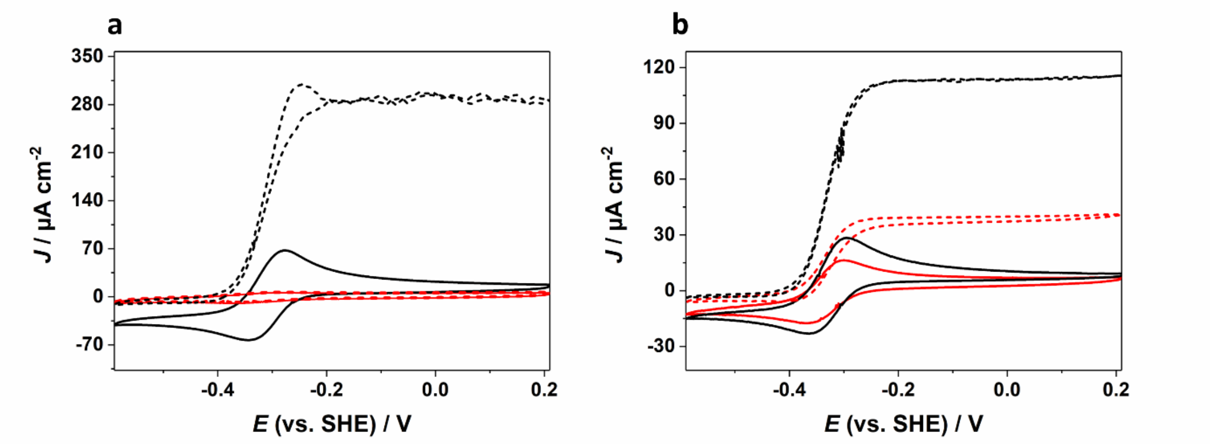

Supplementary Figure 7: Cyclic voltammograms of P(N_3MA-BA-GMA)-vio/*Dv*MF-[NiFe] modified electrodes covered with a GOx/CAT (**a**) or Py_2Ox/CAT (**b**) protection layer measured before (black lines) or after (red lines) long-term measurements as depicted in Figure 3B in the main text under 100 % argon (solid lines) and under 100 % H_2 (dashed lines). Working electrolyte: 0.1 M PB, pH 7.4; scan rates for all measurements: 10 mV s^{-1}. Note that after the long-term measurement using the GOx/CAT protection system, no catalytic response was observed under turnover conditions (dashed red line in **a**) indicating that the hydrogenase was deactivated within the timescale of the long-term experiment. For voltammetry after the long-term experiments the electrodes were transferred into a fresh electrolyte solution to ensure identical conditions for all experiments (note that for the GOx/CAT system the pH value of the electrolyte was considerably lower after the long-term experiment).

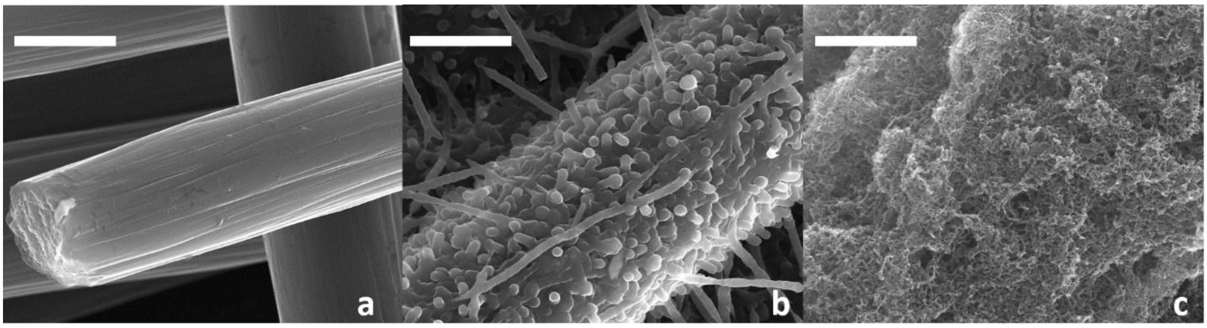

Supplementary Figure 8: Scanning electron micrographs of the bare carbon cloth (**a**), CMF modified carbon cloth (**b**), and CNT/CMF decorated carbon cloth (**c**); scale bar = 5 µm; conditions: **a:**) working distance = 9.2 mm, acceleration voltage = 20 kV; **b**: 9.6 mm, 20 kV; **c:** 8.8 mm, 20 kV. Scale bar = 5 µm.

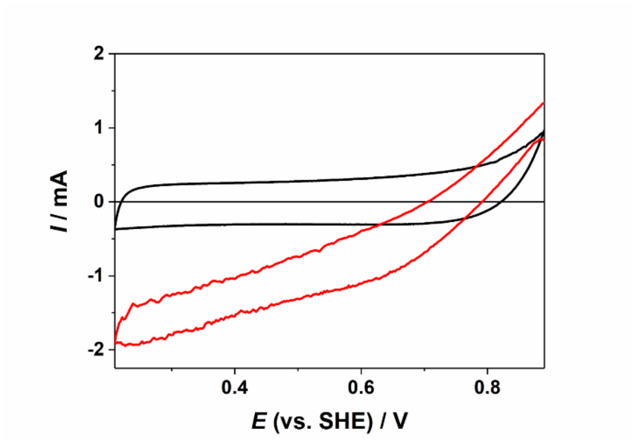

Supplementary Figure 9: Cyclic voltammograms of a CNT/CMF-decorated carbon cloth electrode modified with a HRP layer under Ar and in absence (black line) and presence of 2 mM H_2O_2 (red line); scan rate = 2 mV s^{-1}; working electrolyte = 0.1 M PB, pH 7.4.

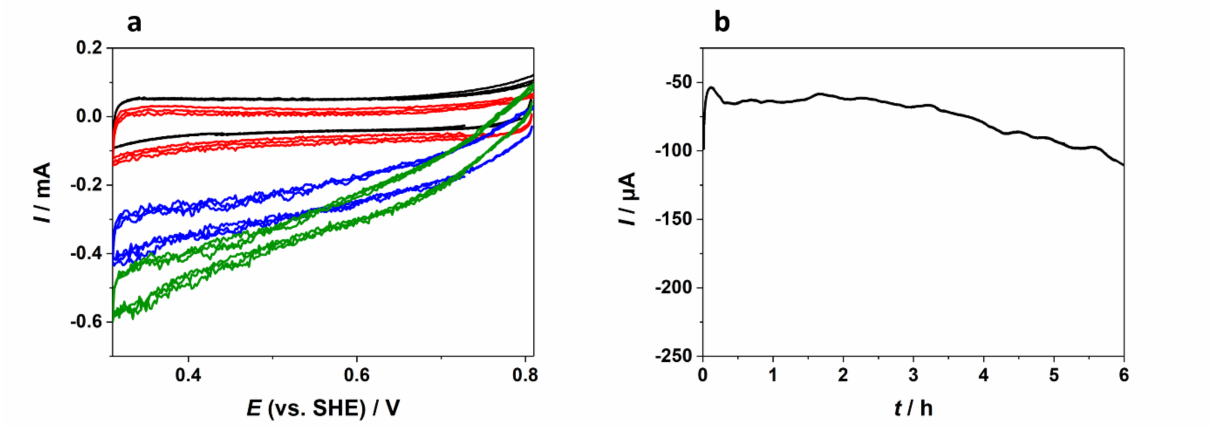

Supplementary Figure 10: Characterization of the GOx/HRP based biocathode. **a:** Dependency of the absolute current of a GOx/HRP modified CNT/CMF carbon cloth electrode on the glucose concentration in solution (0.1 M PB, pH 7.4); Cyclic voltammograms were recorded with a scan rate of 5 mV s^{-1} and with 50 % Ar/50 % O_2 bubbling through the electrolyte; three consecutive potential cycles for each concentration; black line: 0 mM glucose, red line: 1 mM glucose, blue line: 5 mM glucose, green line: 20 mM glucose. Note that the current obtained for 20 mM is close to the currents obtained for 50 mM (Figure 4 in the main text), indicating that saturation is reached at this high glucose concentrations. **b**: chronoamperometric measurements with a GOx/HRP/P(SS-GMA-BA) modified CNT/CMF carbon cloth (the diamine 2,2'-(ethylenedioxy)diethylamine was to crosslink P(SS-GMA-BA)) electrode in 0.1 M PB (pH 7) containing 5 mM glucose with an applied potential of +0.31 V vs. SHE. Note that for the biofuel cell test an additional P(SS-GMA-BA) layer was not employed to facilitate substrate transport and hence to ensure that the bioanode is limiting. However, the stability is comparable to the system depicted in **b**: a biofuel cell test was conducted within ≈3 h.

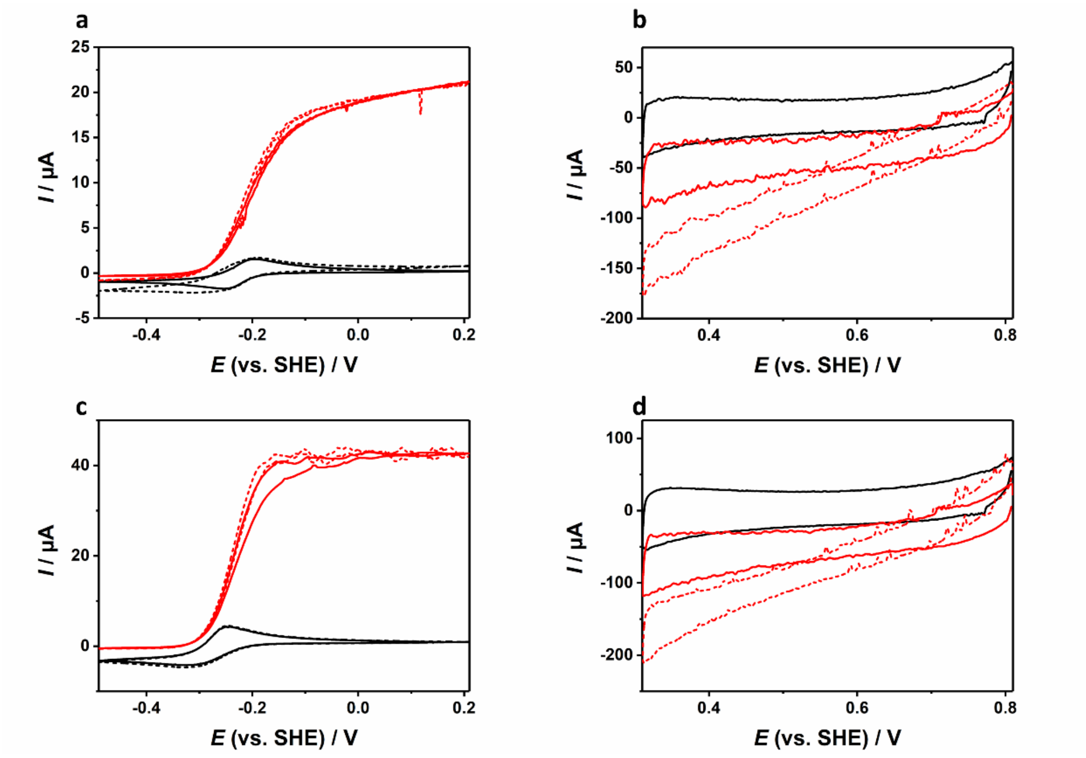

Supplementary Figure 11: Cyclic voltammograms recorded with P(N_3MA-BA-GMA)-vio/H_2ase//P(SS-GMA-BA)/Py_2Ox/CAT bioanodes (H_2ase: **a**: *Dv*MF-[NiFe]; **b**: *Dv*H-[NiFeSe]) and Py_2Ox/HRP-carbon cloth based biocathodes (**b** and **d**) under turnover (red lines) and non-turnover (black lines) conditions before (solid lines) and after (dashed lines) BFC evaluation as depicted in Figure 5 in the main text (two-compartment configuration). **a** and **c**: black lines: 100 % Ar, red lines: 100 % H_2. **b** and **d**: black lines: 100 % Ar, 1 mM glucose; red lines: 100 % O_2, 1 mM glucose. All voltammograms were recorded in 0.1 M PB (pH 7.4) with a scan rate of 10 mV s^{-1}.

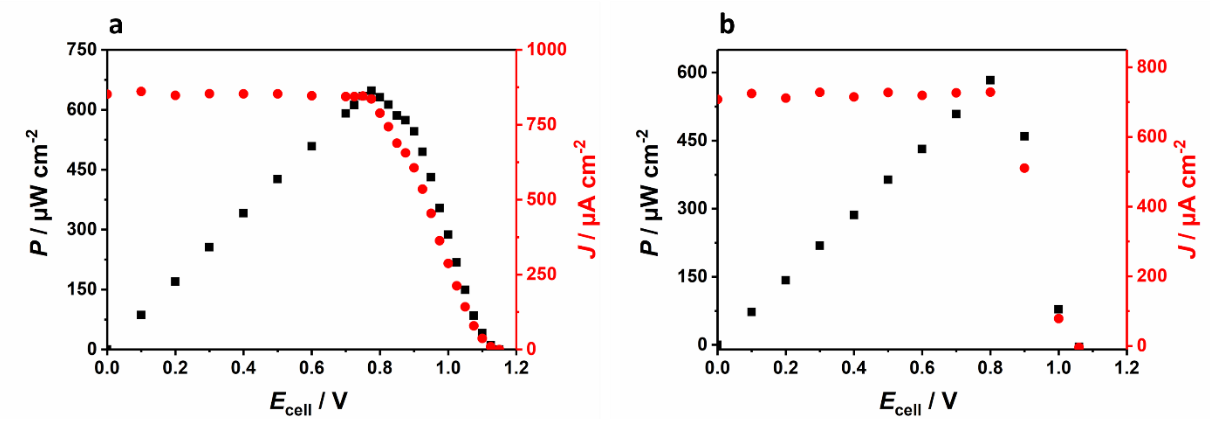

Supplementary Figure 12: Power curves and current densities for P(N_3MA-BA-GMA)-vio/*Dv*H-[NiFeSe]//P(SS-GMA-BA)/Py_2Ox/CAT (bioanode) and Py_2Ox/HRP (biocathode) BFCs in a two-compartment cell containing 0.1 M PB (pH 7.4) and 3 mM (**a**) or 50 mM (**b**) glucose (note that a higher glucose concentration does not lead to an improved performance of the BFC since the anode is limiting in the applied configuration). Characteristic data for **a**: OCV = 1.15 V, J = 830 µA cm^{-2} and P_{max} = 650 µW cm^{-2} at 0.78 V with; **b**: OCV: 1.05 V, J = 730 µA cm^{-2} and P_{max} = 580 µW cm^{-2} at 0.8 V. Gas feeds: bioanode compartment = 100 % H_2/3 % Ar; biocathode compartment = 50 % O_2/50 % Ar.

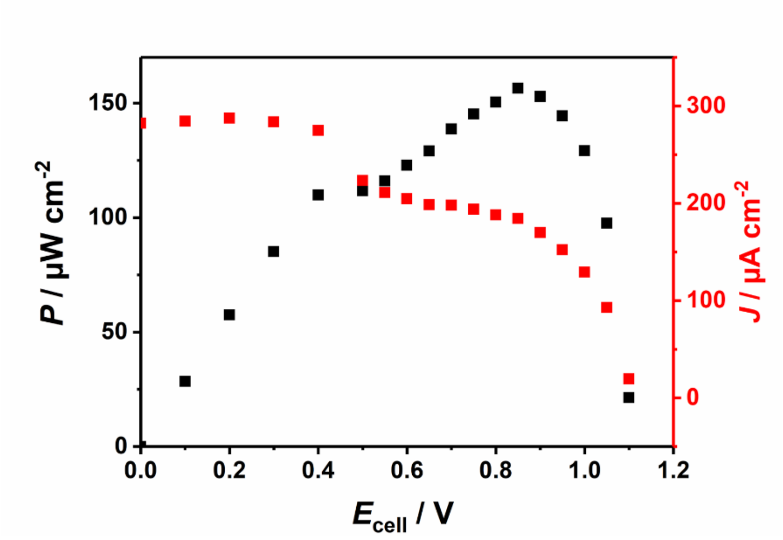

Supplementary Figure 13: Performance of the H_2/glucose(H_2O_2) powered *Dv*H-[NiFeSe]/HRP based BFC in 0.1 M PB (pH 7.4, 3 mM glucose) measured in a one-compartment cell with the polymer double-layer bioanode (P(N_3MA-BA-GMA)-vio/*Dv*H-[NiFeSe]//P(SS-GMA-BA)/ Py_2Ox/CAT) and a Py_2Ox/HRP modified CNT/CMF-carbon cloth biocathode. Characteristics: OCV = 1.15 V; J = 180 µA cm^{-2} and P_{max} = 160 µW cm^{-2} at 0.85 V; gas feed: 97 % H_2/3 % O_2. The jump in J and P at 0.4 V may be attributed to a change at the cathode during the measurement that was started at OCV.

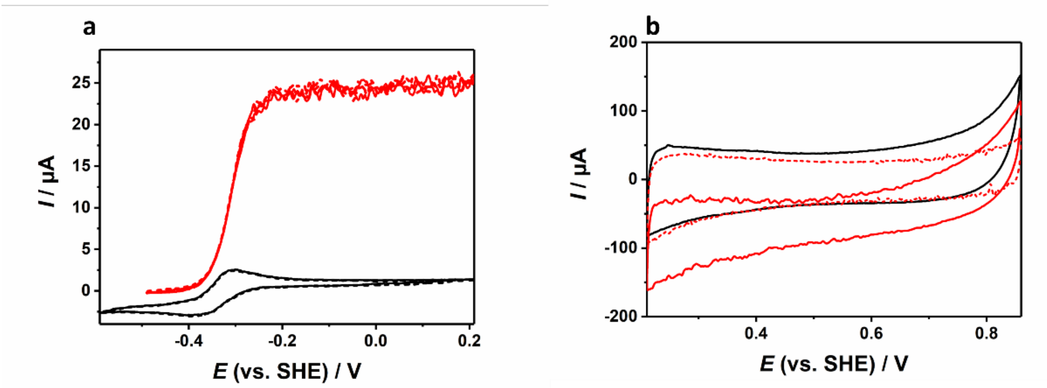

Supplementary Figure 14: Cyclic voltammograms recorded with a P(N_3MA-BA-GMA)-vio/*Dv*H-[NiFeSe]// P(SS-GMA-BA)/Py_2Ox/CAT bioanode (**a**) and a Py_2Ox/HRP-carbon cloth biocathode (**b**) under turnover (red lines) and non-turnover conditions before (solid lines) and after (dashed lines) the BFC test in a one-compartment cell (Supplementary Figure S13). **a**: black lines: 100 % Ar, red lines: 100 % H_2. **b**: black lines: 100 % Ar, 3 mM glucose. All voltammograms were recorded in 0.1 PB (pH 7.4) with a scan rate of 10 mV s^{-1}.

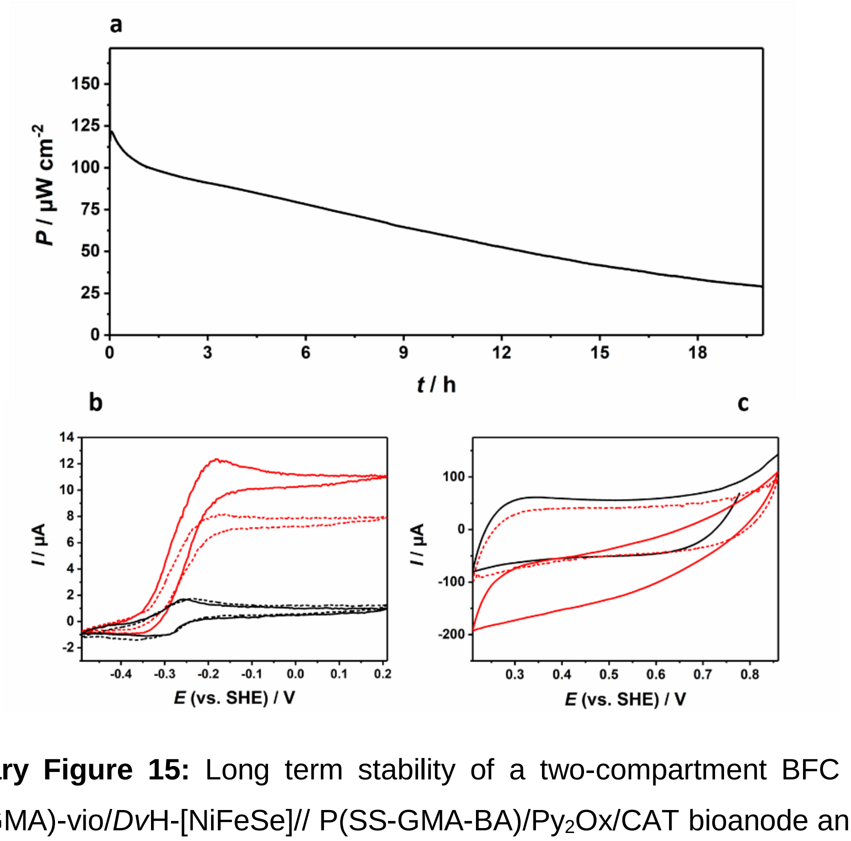

Supplementary Figure 15: Long term stability of a two-compartment BFC comprising a P(N_3MA-BA-GMA)-vio/*Dv*H-[NiFeSe]// P(SS-GMA-BA)/Py_2Ox/CAT bioanode and Py_2Ox/HRP biocathode in 0.1 M PB (pH 7.4) with 3 mM glucose in each compartment and with 97 % H_2/3 % Ar and 50 % O_2/50 % Ar bubbling to the bioanode and biocathode compartment respectively (**a**). Cyclic voltammograms of the bioanode (**b**) and the biocathode (**c**) were recorded in 0.1 M PB (pH 7.4) containg 3 mM glucose before (solid lines) and after (dashed lines) under non-turnover conditions (black lines, **b** and **c**: 100 % Ar) and turn-over conditions (red lines, **b**: 100 % H_2, C: 50 % O_2/50 % Ar).

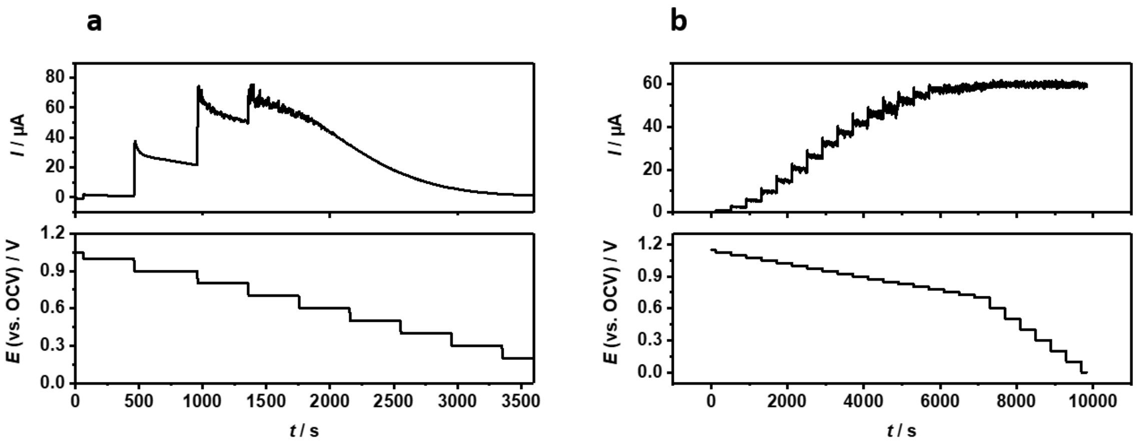

Supplementary Figure 16: Biofuel cell measurement in 0.1 M PB (pH 7.4) with a P(N_3MA-BA-GMA)-vio/DvH-[NiFeSe]//P(SS-GMA-BA)/Py2Ox/CAT bioanode and a Py_2Ox/HRP-carbon cloth biocathode assembled in a two-compartment cell. **a**: the bioanode contained an inactive catalase (H_2O_2 that is produced by Py_2Ox in the presence of O_2 and glucose is not removed at the bioanode). The current (top) drops to zero after ≈3000 s, while applying different loads (bottom) indicating that the formed H_2O_2 destroys the hydrogenase and the polymer matrix (see Supplementary Figure 16). **b**: Current output for a system bearing an active catalase in the protection layer at the [NiFeSe] based bioanode (data correspond to power curve depicted in Supplementary Figure 12A). Stable steady state currents were observed for each load.

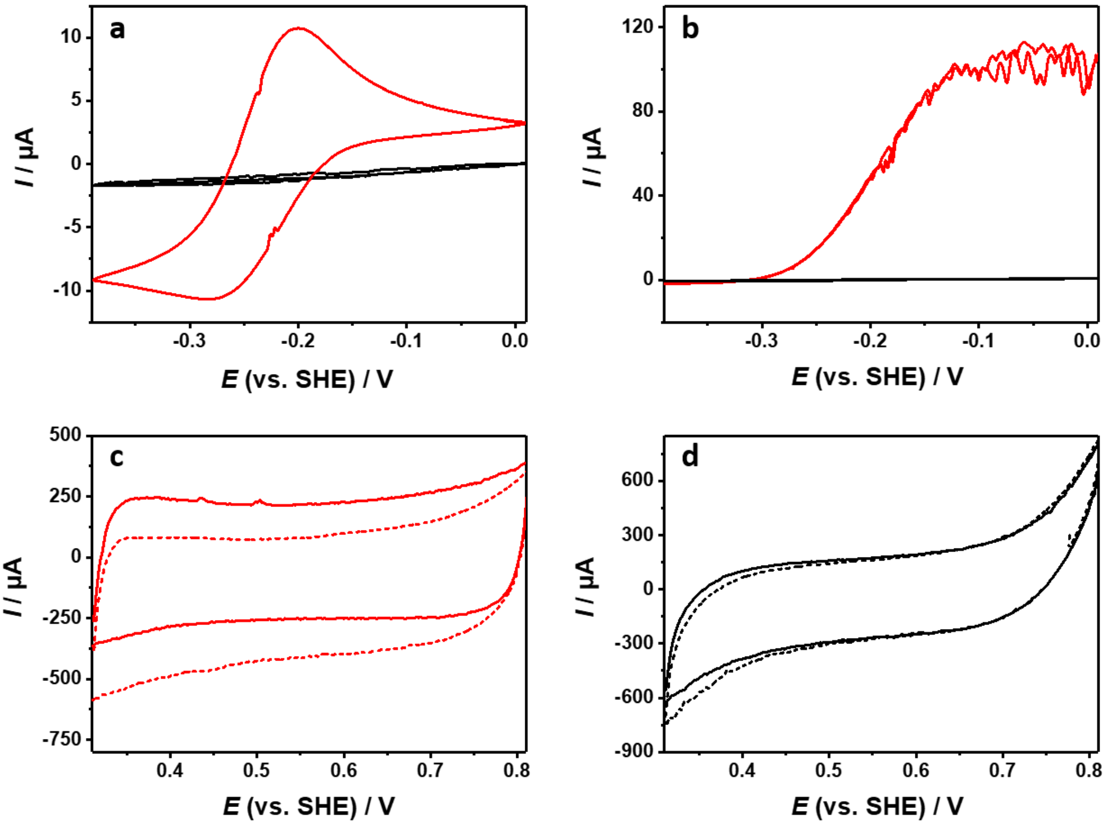

Supplementary Figure 17: Cyclic voltammograms recorded in 0.1 M PB (pH 7.4) with a P(N_3MA-BA-GMA)-vio/*Dv*H-[NiFeSe]//P(SS-GMA-BA)/Py_2Ox/CAT bioanode (**a** and **b**) and a Py_2Ox/HRP-carbon cloth biocathode (**c** and **d**) before (red lines) and after (black lines) a biofuel cell measurement (see Supplementary Figure 15A). The bioanode contained an inactive catalase to demonstrate the effect of H_2O_2 on the bioanode system. **a** and **b**: Cyclic voltammograms recorded with the bioanode under argon (**a**) and H_2 (**b**) bubbling. **c** and **d**: Cyclic voltammograms recorded with the biocathode under argon (solid lines in **c** and **d**) and O_2 (dashed lines in **c** and **d**) bubbling. All voltammograms were recorded with a scan rate of 10 mV s^{-1}.

Supplementary References

1. Ruff, A. *et al.* Protection and Reactivation of the [NiFeSe] Hydrogenase from *Desulfovibrio vulgaris Hildenborough* under Oxidative Conditions. *ACS Energy Lett.* **2,** 964–968 (2017).
